# Plasma proteome profiling of freshwater and seawater life stages of rainbow trout (*Oncorhynchus mykiss*)

**DOI:** 10.1371/journal.pone.0227003

**Published:** 2020-01-03

**Authors:** Bernat Morro, Mary K. Doherty, Pablo Balseiro, Sigurd O. Handeland, Simon MacKenzie, Harald Sveier, Amaya Albalat

**Affiliations:** 1 Institute of Aquaculture, University of Stirling, Stirling, Scotland, United Kingdom; 2 Institute of Health Research and Innovation, Centre for Health Science, University of the Highlands and Islands, Inverness, Scotland, United Kingdom; 3 NORCE AS, Universitetet i Bergen, Bergen, Norway; 4 Lerøy Seafood Group ASA, Universitetet i Bergen, Bergen, Norway; Northwest Fisheries Science Center, UNITED STATES

## Abstract

The sea-run phenotype of rainbow trout (*Oncorhynchus mykiss*), like other anadromous salmonids, present a juvenile stage fully adapted to life in freshwater known as parr. Development in freshwater is followed by the smolt stage, where preadaptations needed for seawater life are developed making fish ready to migrate to the ocean, after which event they become post-smolts. While these three life stages have been studied using a variety of approaches, proteomics has never been used for such purpose. The present study characterised the blood plasma proteome of parr, smolt and post-smolt rainbow trout using a gel electrophoresis liquid chromatography tandem mass spectrometry approach alone or in combination with low-abundant protein enrichment technology (combinatorial peptide ligand library). In total, 1,822 proteins were quantified, 17.95% of them being detected only in plasma post enrichment. Across all life stages, the most abundant proteins were ankyrin-2, DNA primase large subunit, actin, serum albumin, apolipoproteins, hemoglobin subunits, hemopexin-like proteins and complement C3. When comparing the different life stages, 17 proteins involved in mechanisms to cope with hyperosmotic stress and retinal changes, as well as the downregulation of nonessential processes in smolts, were significantly different between parr and smolt samples. On the other hand, 11 proteins related to increased growth in post-smolts, and also related to coping with hyperosmotic stress and to retinal changes, were significantly different between smolt and post-smolt samples. Overall, this study presents a series of proteins with the potential to complement current seawater-readiness assessment tests in rainbow trout, which can be measured non-lethally in an easily accessible biofluid. Furthermore, this study represents a first in-depth characterisation of the rainbow trout blood plasma proteome, having considered three life stages of the fish and used both fractionation alone or in combination with enrichment methods to increase protein detection.

## 1. Introduction

Migratory animals take advantage of seasonally predictable patterns of resource availability and predator abundance and migrate accordingly aiming at maximal survival and to meet their energy demands, though there is often a trade-off between the two [1,2]. For fish, most of these migrations occur within the same water type (i.e. freshwater or seawater), while less than 1% of fish species cross the boundary between freshwater and seawater [3]. This life strategy, known as anadromy for fish that start their life cycle in freshwater to then migrate to seawater, is widespread among several fish families, the most studied of which are the salmonids (salmons, trouts and charrs).

Juvenile anadromous salmonids, called parr, lack the biological traits needed for life in seawater. Upon reaching a threshold size [4], environmental cues, such as changes in photoperiod, water temperature and salinity, trigger hormonal alterations involving their pituitary, thyroid and inter-renal tissues [5]. In turn, these tissues orchestrate a series of simultaneous, yet often independent, changes that preadapt anadromous salmonids to life in seawater [6,7]. These changes are biochemical, such as the increase of gill Na^+^, K^+^ -ATPase activity (NKA), which is the main enzyme involved in ion absorption and secretion and seawater tolerance [8,9]; morphological, such as the transition from dark, rounded parr to a silvery, streamlined phenotype [10]; and behavioural, including the shift from bottom-dwelling, aggressive and territorial parr to pelagic, schooling and downstream migrating [11]. All of these changes are collectively grouped under the term smoltification and the resulting phenotype is known as a smolt. Then, after successfully reaching the ocean, they enter the post-smolt stage.

Extensive literature dating back to the 1950s is available on smoltification, its relationship with salmonid migration, ways to induce it in commercially valuable species, and subsequent seawater performance [6,12,13]. A vast majority of these studies have a clear emphasis on the endocrinology of the process [5,7,14] or on treatments to induce an increase in NKA activity levels [15–17]. However, in the last two decades, and especially after the publication of the Atlantic salmon (*Salmo salar*) and rainbow trout (*Oncorhynchus mykiss*) genomes [18,19], an increasing number of DNA and RNA-based studies have been published on smoltification. Findings include gene expression changes after seawater transfer [20–22], epigenetic modifications [23], quantitative trait loci [24], and gene expression patterns [25–27] associated with the likelihood of steelhead trout to migrate to seawater. At the protein level, studies are restricted to targeted, top-down studies of key protein hormones such as insulin, insulin–like growth factor 1 (IGF-I), growth hormone (GH) and their receptors [8,28,29]. Apart from these, very little proteomic research has been done on smoltification. In this sense, an untargeted, bottom-up protein approach (i.e. proteomics) would have the potential to identify proteins previously unknown to be related to the smoltification process, which could be used as biomarkers in the future [30]. However, so far to our knowledge no proteomic work has been published in relation to the smoltification process.

Shotgun proteomics has been used for the study of proteins in complex biological samples [31]. To this end, liquid chromatography tandem MS (LC-MS/MS) is one of the preferred pipelines used due to its high versatility and high protein identification potential in complex samples. This is achieved due to the physical separation capabilities of liquid chromatography coupled with the ionization capabilities of mass spectrometry [32]. However, LC-MS/MS alone has not sufficient capability to comprehensively analyse complex samples with a high dynamic range such as blood plasma [33]. To increase the coverage of the plasma proteome, samples can be fractionated prior to LC-MS/MS, thus dividing the original sample in less complex subsamples. One approach to increase plasma proteome coverage is to physically separate the proteins present according to their size by 1-dimensional sodium dodecyl sulphate polyacrylamide gel electrophoresis (1-D SDS-PAGE), cutting the gels at specific intervals, protease digesting each gel fraction independently, and analysing each protein digest one by one [34,35]. This approach is known as gel electrophoresis LC-MS/MS (GeLC-MS/MS). However, even after this fractionation, low-abundant proteins might not be detected. Though low in abundance, these proteins can be highly relevant [36]. In order to detect this group of molecules, either a further high-abundant protein depleting step or low-abundant protein enrichment step approaches are needed. Among them, Bio-Rad’s ProteoMiner^™^ enrichment has been shown to significantly improve proteome coverage in blood plasma [37–39].

Blood plasma is a key biofluid for the transport of proteins and peptides to and from tissues, thus containing other tissue proteomes as subsets, making it the single, most complex (with proteins that differ in over 10 orders of magnitude in abundance; from milligrams to pictograms per millilitre [33]) and informative proteome [40–42]. Plasma protein studies have succeeded in discovering biomarkers for disease [43–45], growth [46,47], stress [48,49], exposure to water contaminants [50,51], or doping [52], amongst many others. Therefore, it is highly likely that more protein biomarkers for the smoltification and seawater adaptation processes may be discovered in blood plasma.

The aim of this study was to characterise proteome changes in blood plasma of rainbow trout, associated to the smoltification process. Its objectives were to 1) provide an in-depth characterization of the plasma proteome of rainbow trout, 2) make this plasma proteome publicly available, and 3) discover new candidate biomarkers to complement current seawater-readiness evaluation tests in rainbow trout.

## 2. Materials and methods

### 2.1. Ethics

Experimental work was in accordance to Directive 2010/63/EU guidelines and ethically reviewed, approved and registered by the NARA, by the AWERB (088) at the University of Stirling (UK) and by the ethical review body of the University of the Highlands and Islands. Access to collection sites was approved by the co-author HS, technical manager of Lerøy Seafood Group ASA.

### 2.2. Fish and rearing conditions

Juvenile rainbow trout (AquaGen) with an initial weight of 78 ± 16.7 g were used in this experiment. Fish were fed *ad libitum* using a standard commercial dry diet (Skretting AS) from automatic feeders according to temperature and fish size. Fish were kept indoors in tanks equipped with timer-controlled LED lights in a rainbow trout facility from Lerøy Vest AS (Bjørsvik, Hordaland, Norway. 60°37'58.0"N 5°29'43.8"E). The fish were kept at natural temperature, water flow at 0.4 L/kg/min and O_2_ was above 80% in the outlet. Freshwater was supplied from Lake Husdalsvatnet and seawater from Fjord Bjørsvika.

The present experiment was carried out on a subset of samples generated in a previous study [53]. Therein, details on environmental water temperature, experimental design (initially, 110 fish per tank in 8 tanks), and samplings (6 fish per tank per sampling during the freshwater stage (10 samplings) and a total of 306 fish from 4 tanks at the endpoint sampling in seawater) can be found in [53].

### 2.3. Sampling

Lethal samplings were conducted in freshwater on the 3^rd^ of March and 11^th^ of May and in seawater on the 14^th^ of September 2016.

Fish were quickly dip-netted out of the tanks and euthanized by lethal overdose of isoeugenol (AQUI-S), following Directive 2010/63/EU guidelines. For each fish, weight and length were recorded. Blood was extracted immediately after euthanasia using heparinised syringes and centrifuged at 3,500g for 10 min to obtain plasma, which was frozen at -80°C. The first gill arch was dissected out and preserved at -80°C in SEI buffer (Sucrose 250mM, Na_2_EDTA 10mM, Imidazole 50mM (all Sigma-Aldrich)).

### 2.4. Gill NKA activity

NKA activity was measured according to McCormick’s methodology, which couples the hydrolysis of ATP to the enzymatic production of NAD^+^ through the involvement of the enzymes pyruvate kinase and lactate dehydrogenase, and uses the NKA inhibitor ouabain to trace the baseline [54]. Kinetic assay readings were carried out at 340 nm for 10 min (60 cycles) at 25°C in a in a Sunrise-basic (Tecan) spectrophotometer. Total amount of protein in the homogenate was analysed using a bicinchoninic acid (BCA) assay run in triplicate. NKA values were determined as the ouabain sensitive fraction of the ATP hydrolysis, expressed as μmol ADP mg protein^−1^ hour^−1^.

### 2.5. Sample pools for proteomic analysis

Three pools of untreated (non-enriched) plasma were made using equal amounts of protein per sample (measured by BCA): Parr pool, Smolt pool and Post-smolt pool (Table 1; name of pools is capitalized hereafter while name of developmental stage is not). Enough volume of pooled plasma to carry out analysis of both untreated plasma and plasma enriched using low-abundant protein enrichment technology (explained in the next section) was needed. Samples were selected independently of their tank of origin, according to the following criteria. The Parr pool was made using 17 fish sampled in March (3^rd^ March) that presented NKA values below 4 μmol ADP mg protein^−1^ hour^−1^. The Smolt pool using 18 fish sampled during the smolt window (11^th^ May) that presented NKA values above 6 μmol ADP mg protein^−1^ hour^−1^, a value that is considered indicative of osmocompetence in seawater for rainbow trout and therefore of fish having entered their smolt phase [55,56]. Finally, the Post-smolt pool was made using 12 fish sampled at the end-point sampling in seawater (14^th^ September), 9 weeks after seawater transfer, that presented a condition factor above 1.50 g cm^-3^, thus avoiding the growth-stunted fish, which are a phenotype that commonly appears after seawater transfer, characterized by high mortalities, stunted growth and a decrease in condition factor [53].

**Table 1:** Measurements in fish used for plasma pools (values ± standard error).

Pool	Sampling	Length (cm)	Weight (g)	Fulton index	NKA activity
**Parr**	3^rd^ March	18.5 ± 0.39^c^	85.9 ± 5.36^c^	1.3 ± 0.02^b^	1.9 ± 0.26^b^
**Smolt**	11^th^ May	22.2 ± 0.35^b^	147.6 ± 6.37^b^	1.3 ± 0.02^b^	9.9 ± 0.60^a^
**Post-smolt**	14^th^ September	30.2 ± 0.40^a^	434.5 ± 17.08^a^	1.6 ± 0.02^a^	3.0 ± 0.71^b^

Fulton index is measured in g cm^-3^. NKA activity is measured in μmol ADP mg protein^−1^ hour^−1^. Significant differences (p<0.05) are indicated with different superscript letters.

Admittedly, sample pooling is a controversial strategy when used to draw biological conclusions, with studies advocating for [57–59] and against it [60–62]. A limitation of pooling is that proteins detectable in only a few samples are seldom not detectable in pools due to a dilution effect [63,64] but in the present study, a low-abundant protein enrichment step addressed this problem. Moreover, there is the concern that sample pooling may significantly reduce statistical power [63]. However, studies dedicated to studying the effects of sample pooling in proteomics conclude that pooling designs have statistical power almost matching that of separately analysed samples [61–63,65]. Indeed, they showed that for a majority of the proteins, protein expression in a pool matches the mean expression of the individual biological replicates in it. Pooling allows to perform experiments when biological material per sample is limited [66,67], and when sample processing and analysis are excessively time-consuming and expensive [57], as was the case here. In this circumstances, rather than using a small number of individually analysed biological replicates, resulting in low power and complicating the detection of significant differences, a pooled design is more appropriate [61–63,65]. Since pooling makes dominant differences and similarities between groups easily detectable due to an inherent reduction in biological variation, it is an especially appropriate strategy to study populations instead of individuals, such as in biomarker studies or when performing a broad characterization of a sample type [61,68].

**Fig 1:**
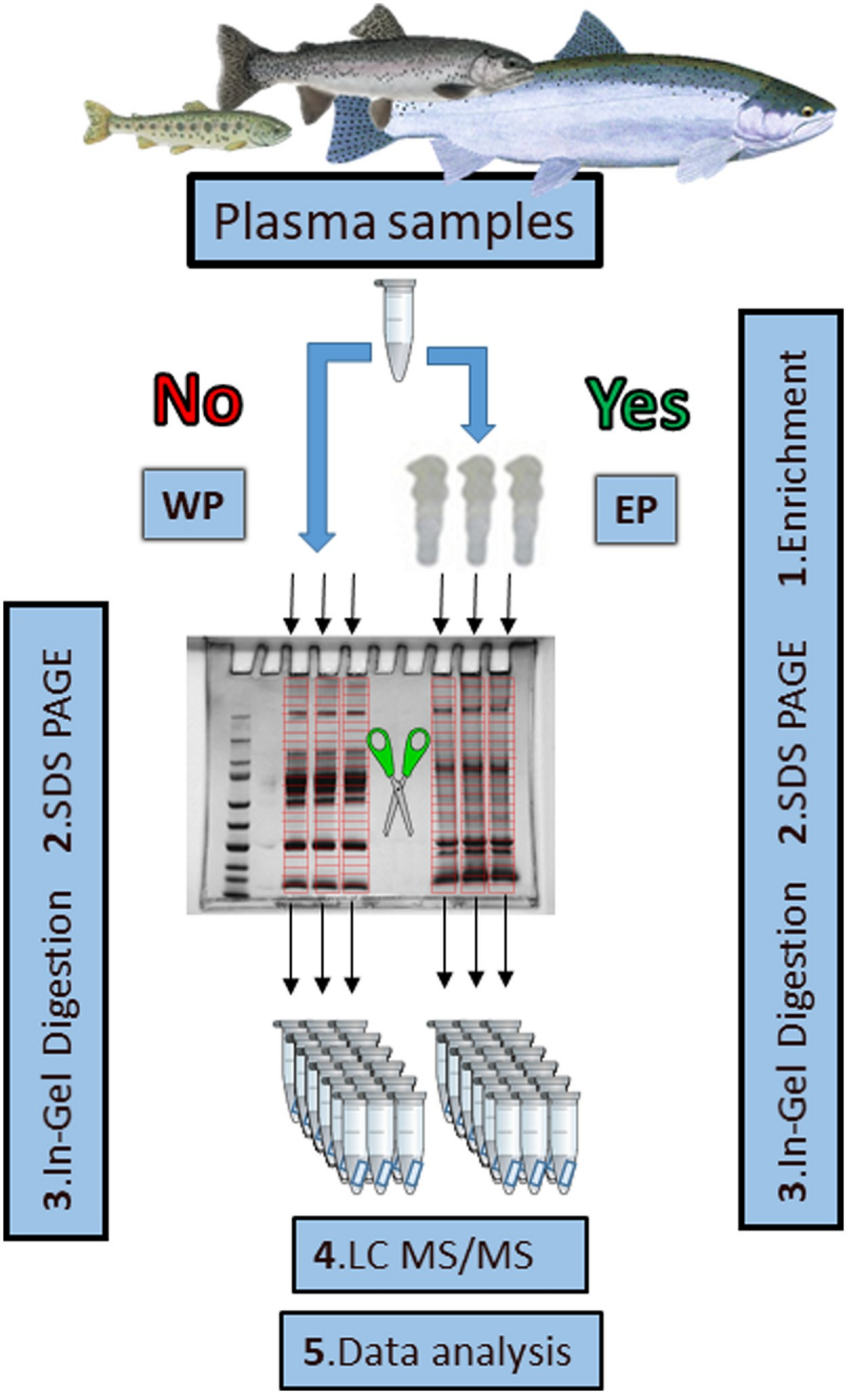
Methodological workflow. Scissors indicate fractionation and red divisions indicate gel cuts (done equally for each lane). EP: Enriched plasma, WP: Whole plasma.

An overview of the full methodological workflow used in the present experiment from this point onwards is provided in Fig 1.

### 2.6. Low-abundance proteins enrichment

Knowing the protein concentration of plasma pools (measured by BCA), enriched plasma (EP) was prepared using ProteoMiner^™^ Protein Enrichment Small-Capacity Kit (Bio-Rad) in triplicates on each of the pools (10 mg of protein) following the manufacturer’s instructions and increasing the sample binding time to 3 h.

### 2.7. Analysis of proteins by GeLC-MS/MS

#### 2.7.1. 1-D SDS-PAGE analysis

For each plasma pool, 10 μg of plasma (EP or whole plasma (WP)) were analysed in triplicates in order to expand protein identifications and to account for technical noise during data analysis [63,69]. Samples were mixed in reducing buffer (13.1 mM Tris—pH 6.8, 2.63% v/v Glycerol, 0.42% v/v sodium dodecyl sulfate (SDS), 0.243% v/v bromophenol blue and 163.5 mM dithiothreitol (DTT)), heated up to 95°C for 5 min and centrifuged at 2,000 g for 30 s. Reduced lysates were loaded into a 1-D SDS polyacrylamide gel (4–15%, Mini-PROTEAN TGX, BIO-RAD) with a protein ladder reference (5μl, BenchMark, 10-220kDa, ThermoFisher Scientific). Gels were run using a Mini PROTEAN Tetra Cell System (Bio-Rad) at 200 V (400 mA) for around 50 min. Protein bands were fixed and stained with SimplyBlue Safestain (Thermo Fisher Scientific) following the manufacturer’s instructions and destained overnight in MilliQ water (Millipore, Merck) at RT. 1-D SDS-PAGE pictures were taken using an inGenius LHR Gel Imaging System (SynGene) and band densitometry data was obtained using GeneTools software version 4.3.8 (SynGene). Profile height (i.e. band intensity) to relative mobility (R_f_) data was imported into R and plotted using ggplot2 package [70].

#### 2.7.2. In-gel digestion

On the next day, each gel lane was cut in 24 gel plugs of 3–4 mm in a laminar flow fume cabinet and fractions were stored in separate 1.5 ml tubes. Destain solution (100 μl) (50% 100mM ammonium bicarbonate (Ambic, Sigma-Aldrich) and 50% acetonitrile (ACN, Fisher Chemical)) was added to each plug and incubated at 37°C for 10 min in a thermoblock. The liquid was discarded and replaced with another 100 μl of destain solution and incubated again for 10 min. After discarding the liquid, 50 μl of 10 mM dithiothreitol (DTT, Bio-Rad) was added to each sample to reduce proteins. After a 30 min incubation at 37°C, the liquid was discarded and the plugs were alkylated with 50 μl of 55 mM iodoacetamide (IAA, GE Healthcare) for 30 min at 37°C. After discarding the liquid, 50 μl of 100% ACN was added to each tube and incubated for 15 min at 37°C. Then, the ACN was removed and the gels plugs were air dried at RT for 10 min before adding 50 μl of trypsin (Roche, 0.01 mg/ml in 10% acetic acid and 45 mM Ambic) and incubating it at 37°C. After 30 min, an extra 20 μl of 50 mM Ambic was added to each tube and left overnight. Then, 70 μl of 100% ACN was added to each tube and incubated at RT for 15 min with shaking. The liquid was transferred to new tubes, while 50 μl of 97.5% ACN and 2.5% formic acid was added to each gel plug and incubated at RT for 15 min with shaking to extract any remaining peptides. This liquid was added to the corresponding tube and the gel plugs were discarded. Finally, the trypsin digests were dried using a vacuum drier (Savant DNA SpeedVac 110, Thermo Scientific).

#### 2.7.3. LC-MS/MS analysis

Tryptic digests were analysed with a LTQ-Orbitrap XL LC−MSn mass spectrometer (Thermo) equipped with a nanospray source and coupled to an Ultra High Pressure Liquid Chromatographer system (Waters nanoAcquity). Initially, 5 μL of sample resuspended in ultrapure water were loaded, desalted and concentrated in a BEH C18 trapping columns (Waters) with the instrument operated in positive ion mode. The peptides were then separated on a BEH C18 nanocolumn (1.7 μm, 75 μm × 250 mm, Waters) at a flow rate of 300 nL/min using an ACN/water gradient; 1% ACN for 1 min, followed by 0−62.5% ACN during 21 min, 62.5− 85% ACN for 1.5 min, 85% ACN for 2 min and 100% ACN for 15 min.

MS spectra were collected using data-dependent acquisition in the m/z range 400−2,000 using a precursor ion resolution of 30,000, following which individual precursor ions (top 5) were automatically fragmented using collision induced dissociation with a relative collision energy of 35%. Dynamic exclusion was enabled with a repeat count of 2, repeat duration of 30 s and exclusion duration of 180 s.

The mass spectrometry proteomics data have been deposited to the ProteomeXchange Consortium via the PRIDE partner repository with the dataset identifier PXD016570.

#### 2.7.4. LC-MS/MS data analysis and protein identification

Mass spectrometry data was analysed using Progenesis QIP (Nonlinear Dynamics). WP and EP datasets were analysed independently following the ‘fractionation experiment’ analysis. Two pairwise comparisons were performed for WP datasets (i.e. Parr (WP) vs. Smolt (WP), Smolt (WP) vs. Post-smolt (WP)) and two more for EP datasets (i.e. Parr (EP) vs. Smolt (EP), Smolt (EP) vs. Post-smolt (EP)). The initial search parameters allowed for a single trypsin missed cleavage, carbamidomethyl fixed modification of cysteine residues, oxidation of methionine (variable), acetylation of N-terminal peptides, a precursor mass tolerance of 10 parts per million, charge of deconvoluted ions of over 1, a fragment mass tolerance of ±0.5 Da, and FDR of 0.01.

After normalization, the Hi3 (Top3, using the three most abundant peptides) method was used for protein quantification [71]; therefore a minimum of 3 peptides was required for quantification. Moreover, only those proteins identified based on at least one unique peptide (a peptide that can only be matched to a single protein in the underlying database) were quantified. Statistical differences were tested by ANOVA in Progenesis QIP. To be considered differentially abundant proteins (DAPs), a q-value below 0.05 (q < 0.05) and a fold change (FC) bigger than 2 was required. However, since the sole purpose of EP was to improve the coverage of WP, proteins quantified in WP were not statistically tested in EP.

Peptide sequences were matched to a database search against the *Oncorhynchus mykiss* SwissProt database, which was downloaded from MASCOT [downloaded in August 2018] and loaded into Progenesis QIP. Those identified as ‘uncharacterised’ in the rainbow trout genome were sequentially blasted against the Atlantic salmon (actinopterygii, salmoniformes, salmonidae. Genome assembly: ICSASG_v2), zebrafish (*Danio rerio*, actinopterygii, cypriniformes, cyprinidae. Genome assembly: GRCz11), and human (*Homo sapiens*, actinopterygii, primates, hominidae. Genome assembly: GRCh38.p13) SwissProt databases, in this order of preference. Only homologies of E-value lower than 0.01 were accepted as valid.

### 2.8. Gene ontology (GO) analysis

GO of biological process, cellular component and molecular function was performed to compare the dataset of unique WP proteins with the dataset of unique EP proteins in order to identify possible differences in protein affinity between the two methods. Analysis was performed using STRAP v. 1.5. [72].

### 2.9. Further data analysis and representation

Data representation was carried out using Microsoft Excel 2013 or R statistical software and R package ggplot2.

One-way ANOVA was performed to test for differences in morphometric measures and NKA activity among developmental stages. Data was transformed by either natural logarithm or square root to satisfy the normal distribution and homogeneity of variance assumptions, tested with the Shapiro and Bartlett tests, respectively. Significant comparisons (p < 0.05) were followed by Tukey’s posthoc test to identify different developmental stages.

Linear relationship among variables was determined by linear regression using the QR method. Significance values (p<0.05) were obtained by testing the null hypothesis: the slope of the least squares linear fit to the data is equal to 0.

Principal component analysis (PCA) and data representation were carried out using R and ‘ggbiplot’ package [73]. Ellipses show 68% of Normal probability for each group.

Mean abundance per condition was plotted for DAPs in heatmap form scaling abundance by protein (i.e. by row).

## 3. Results

### 3.1. Characterisation of rainbow trout plasma according to GeLC-MS/MS alone or in combination with protein enrichment technology

#### 3.1.1. Detected proteins

A total of 48,196 peptides were detected in WP (S1 File), which were mapped onto 2,784 rainbow trout proteins (S2 File). Of these, 1,495 met the requirements for reliable identification and quantification (i.e. quantified proteins; identified based on at least 3 peptides and at least 1 unique peptide). Similarly, for EP, 48,921 peptides were detected (S3 File), which were mapped onto 1,892 rainbow trout proteins and 1,292 were quantified (S4 File). Interestingly, the number of unique peptides found for a particular protein was higher for more abundant proteins (S1 Fig) and significantly correlated with the protein mass and length (S2 Fig).

**Fig 2:**
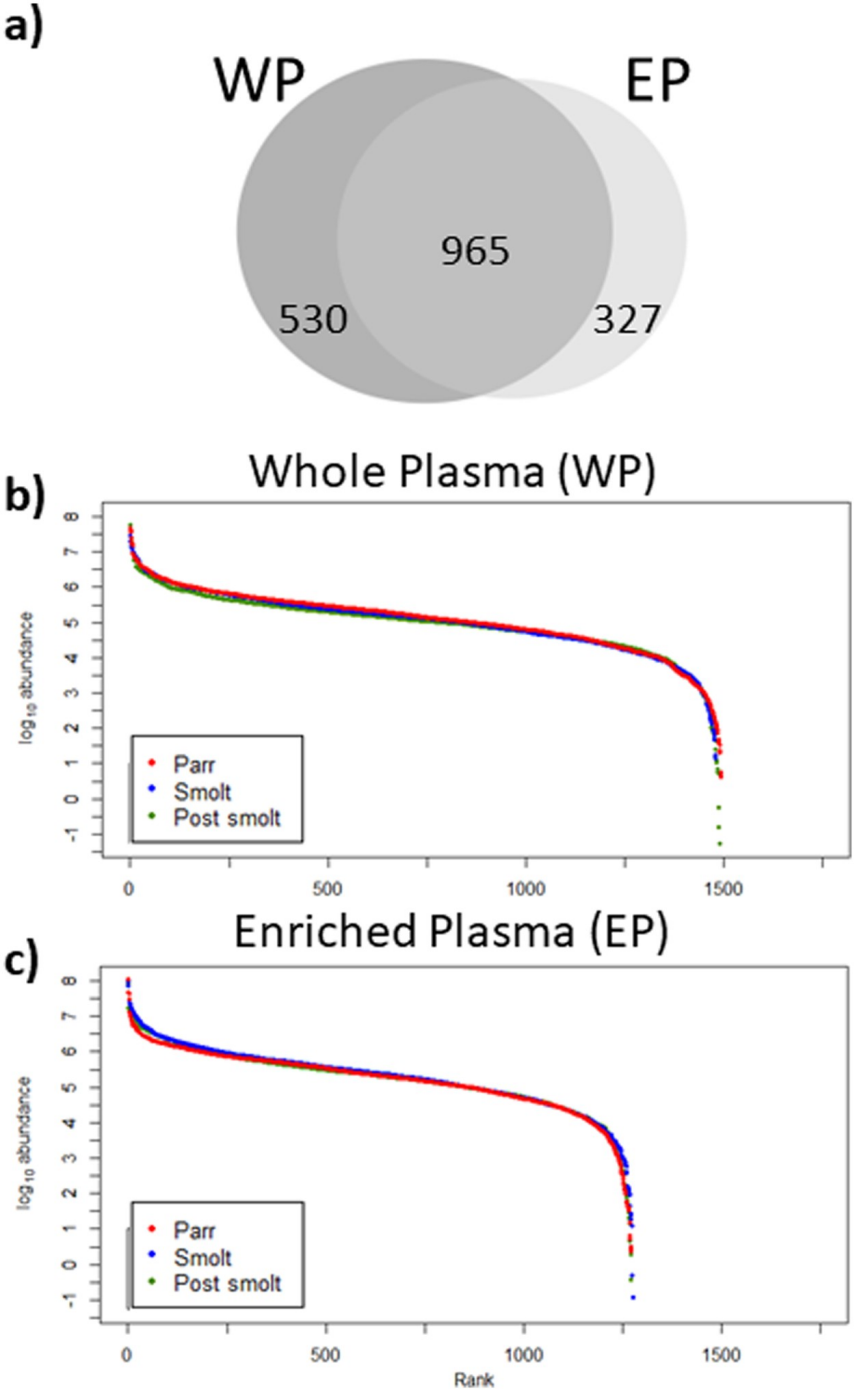
Quantified proteins in whole (WP) and enriched plasma (EP). Venn diagram of proteins quantified in WP and EP (a). Dynamic range of proteins quantified in WP (b) and in EP (c). Rank indicates order of proteins in each condition, from most to least abundant.

Comparison of WP and EP datasets revealed that 965 proteins were detected by both strategies, these being a majority (52.96%). Another 530 (29.01%) were only found in WP samples and 327 (17.95%) were only detected after enrichment—only in EP (Fig 2a). These differences between WP and EP were already apparent when inspecting the protein profiles by 1-D SDS-PAGE, with EP missing some of the very intense bands of WP and having bands that could not be visualized in WP (S3 Fig). For both WP and EP, samples corresponding to the three tested developmental conditions followed a similar trend of dynamic ranges, indicating that the quantitative distribution of the quantified proteins was comparable in Parr, Smolt and Post-smolt pools. For both WP and EP, the quantified proteins presented abundances that covered 9 orders of magnitude (Fig 2b and 2c).

To facilitate comparison with other published or future studies: 2,784 proteins would have been identified based on a minimum of one peptide, 2,534 on a minimum of two peptides, 1,733 based on a minimum of one unique peptide and 885 on a minimum of two unique peptides.

#### 3.1.2. Enrichment correlations

To test which protein physicochemical properties control the change in protein abundance due to enrichment (increase of low-abundant proteins and decrease of high-abundant proteins), correlations between the FC in abundance of a specific protein from WP to EP with the protein’s mass, length and abundance in WP were tested.

**Fig 3:**
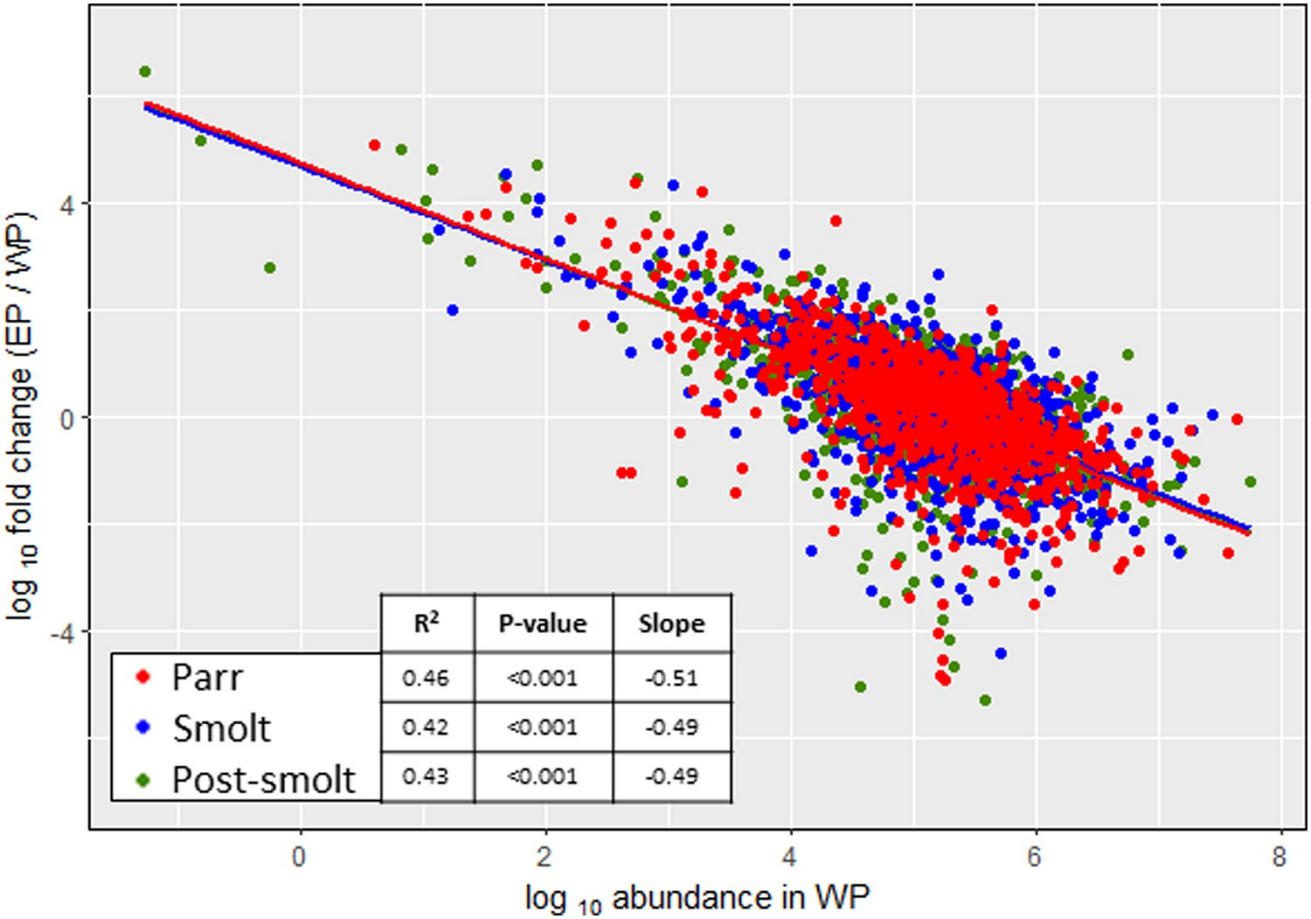
Effects of enrichment. Correlation between the fold change from whole (WP) to enriched plasma (EP) and original abundance in WP for each protein. Points indicate individual samples and lines indicate linear regression fit.

Results indicate that neither protein mass (Parr: r^2^ = 1*10^−4^, p-value = 0.74; Smolt: r^2^ = 1*10^−4^, p-value = 0.76; Post-smolt: r^2^ = 3*10^−4^, p-value = 0.62) nor length (Parr: r^2^ = 1*10^−4^, p-value = 0.75; Smolt: r^2^ = 1*10^−4^, p-value = 0.76; Post-smolt: r^2^ = 3*10^−4^, p-value = 0.62) were correlated with enrichment. However, a pronounced significant correlation was present between the FC of enrichment and the original abundance of the protein in WP (Fig 3, Parr: r^2^ = 0.46, p-value < 0.001; Smolt: r^2^ = 0.42, p-value < 0.001; Post-smolt: r^2^ = 0.43, p-value < 0.001). The low-abundant protein enrichment resulted in the abundance/detectability of low-abundant proteins being increased by up to almost 3 million times and that of high-abundant proteins decreased over 200,000 times.

#### 3.1.3. GO of WP and EP unique proteins

General GO analysis revealed no differences between proteins quantified exclusively in WP or in EP pools regarding biological process, cellular component or molecular function, as there were no exclusive categories to either WP or EP (S4 Fig).

#### 3.1.4. PCA

**Fig 4:**
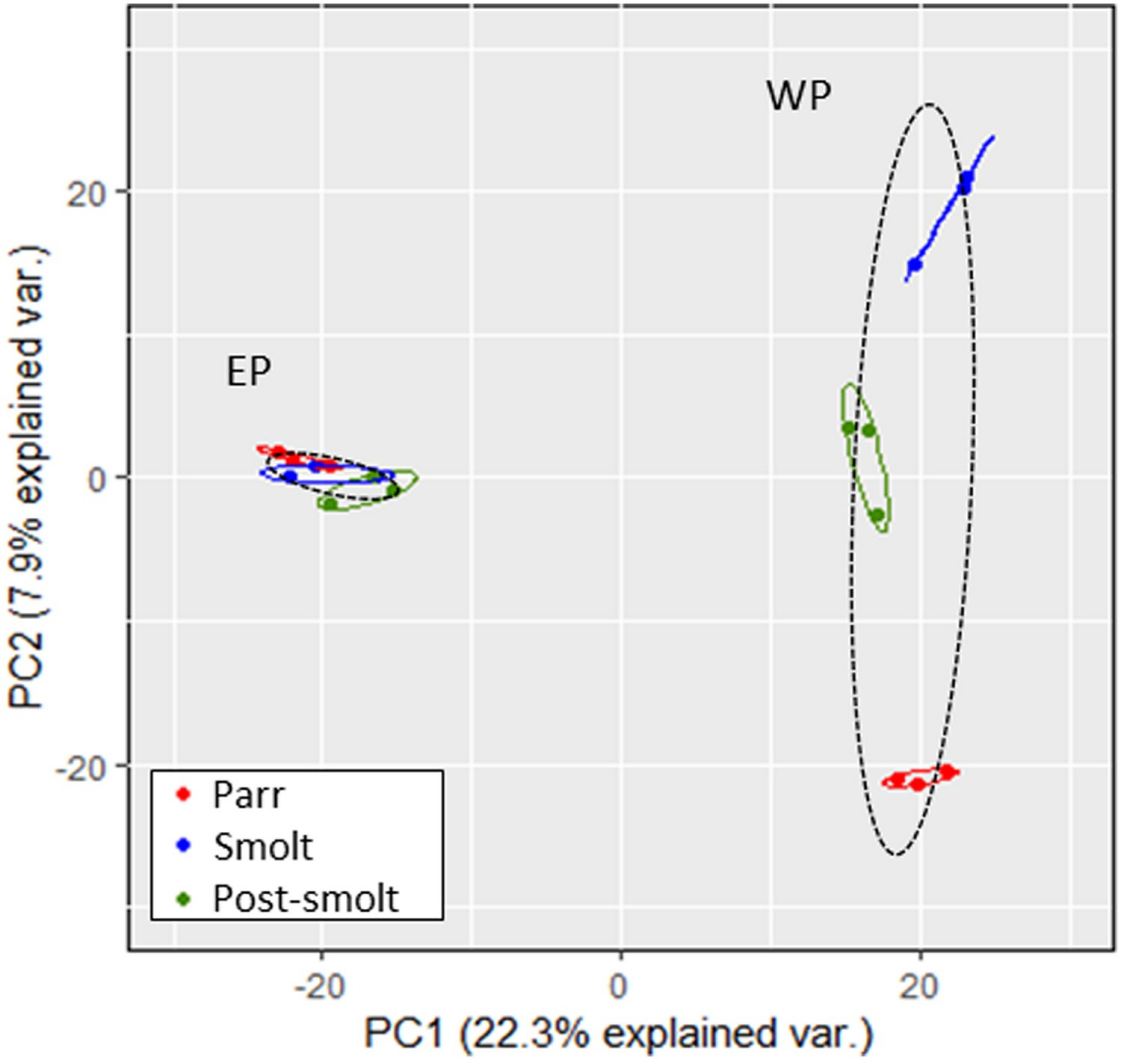
Principal components analysis of quantified parr, smolt and post-smolt rainbow trout plasma proteins. Points indicate individual replicates. Ellipses show 68% Normal probability for each developmental stage (colours) or enrichment strategy (dashed).

PCA was used to visualize the relationship between replicates, the dissimilarity among developmental conditions, and the effects of the enrichment strategy. The first two components of the PCA explained 30.2% of the variation between samples. A clear distinction between the three tested developmental conditions was possible in WP samples, mainly due to PC2, but not in EP samples (Fig 4). The distinction between WP and EP was very clear, mainly driven by PC1.

### 3.2. Characterisation of rainbow trout plasma according to developmental stage

#### 3.2.1. Most abundant proteins

**Fig 5:**
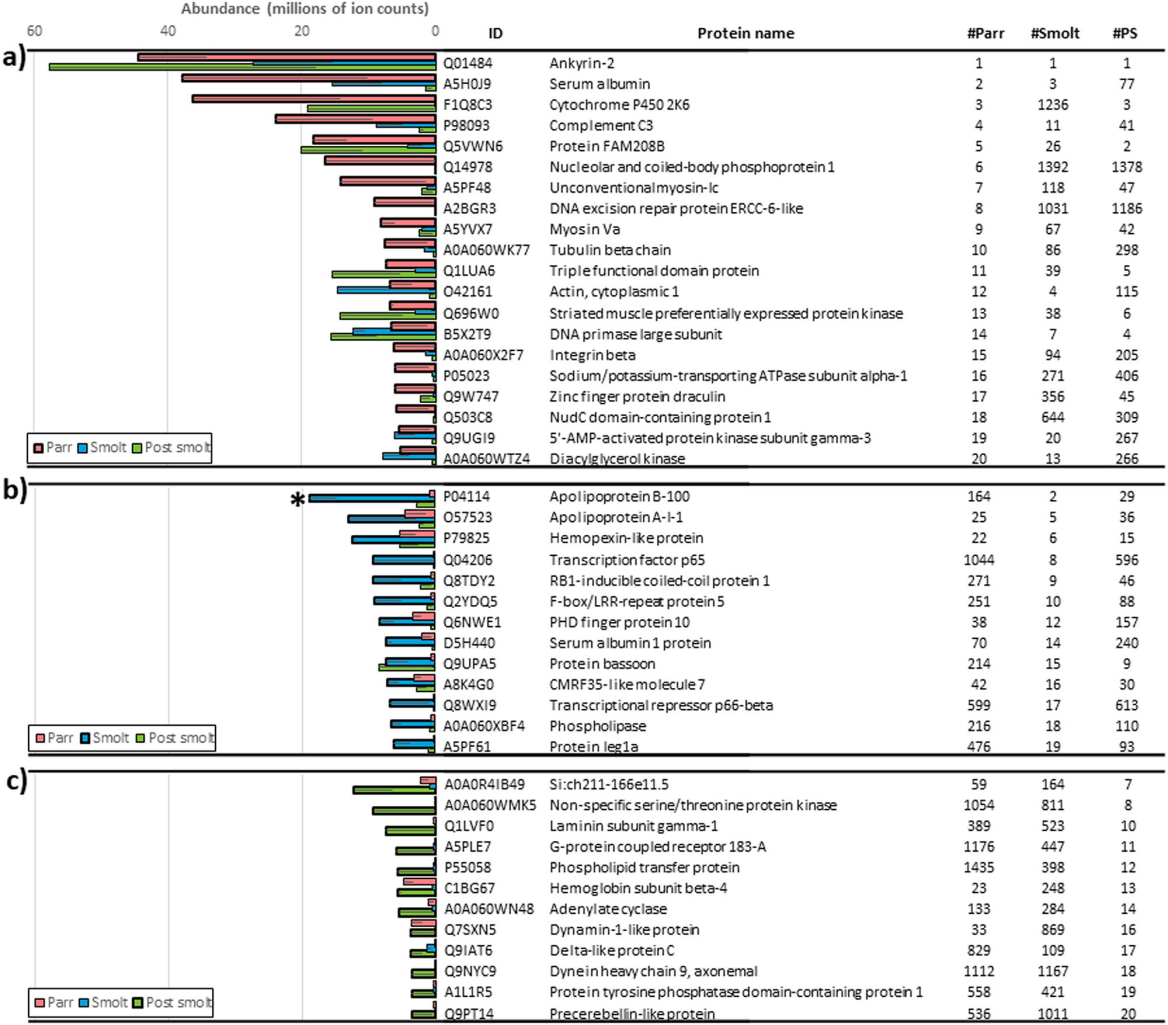
Most abundant proteins in rainbow trout plasma. Top 20 most abundant proteins in Parr (a), Smolt (b) and Post-smolt pools (c) of rainbow trout whole plasma. ‘ID’ indicates the UniProt accession numbers of the proteins. ‘#’ columns indicate the rank in terms of abundance of each protein in each of the three tested developmental stages. ‘*’ indicates overall significant differences among developmental conditions (three plasma pool replicates per developmental condition) as determined by ANOVA. Error bars indicate standard error. PS: Post smolt.

Across all life stages, the most abundant proteins were ankyrin-2, DNA primase large subunit, actin, serum albumin, apolipoproteins, hemoglobin subunits, hemopexin-like proteins and complement C3. However, with the exception of Ankyrin-2, which was invariantly the most abundant protein in all three tested conditions, and DNA primase large subunit, which was consistently among the top 20 in all three conditions, the list of top 20 most abundant proteins was highly variable among Parr, Smolt and Post-smolt pools (Fig 5). Nevertheless, only one of these high-abundant proteins, P04114 (Apolipoprotein B-100), presented significant differences between conditions. Since Proteominer^™^ was used to study low abundant proteins, EP it was not considered in this section.

#### 3.2.2. Differentially abundant proteins

**Fig 6:**
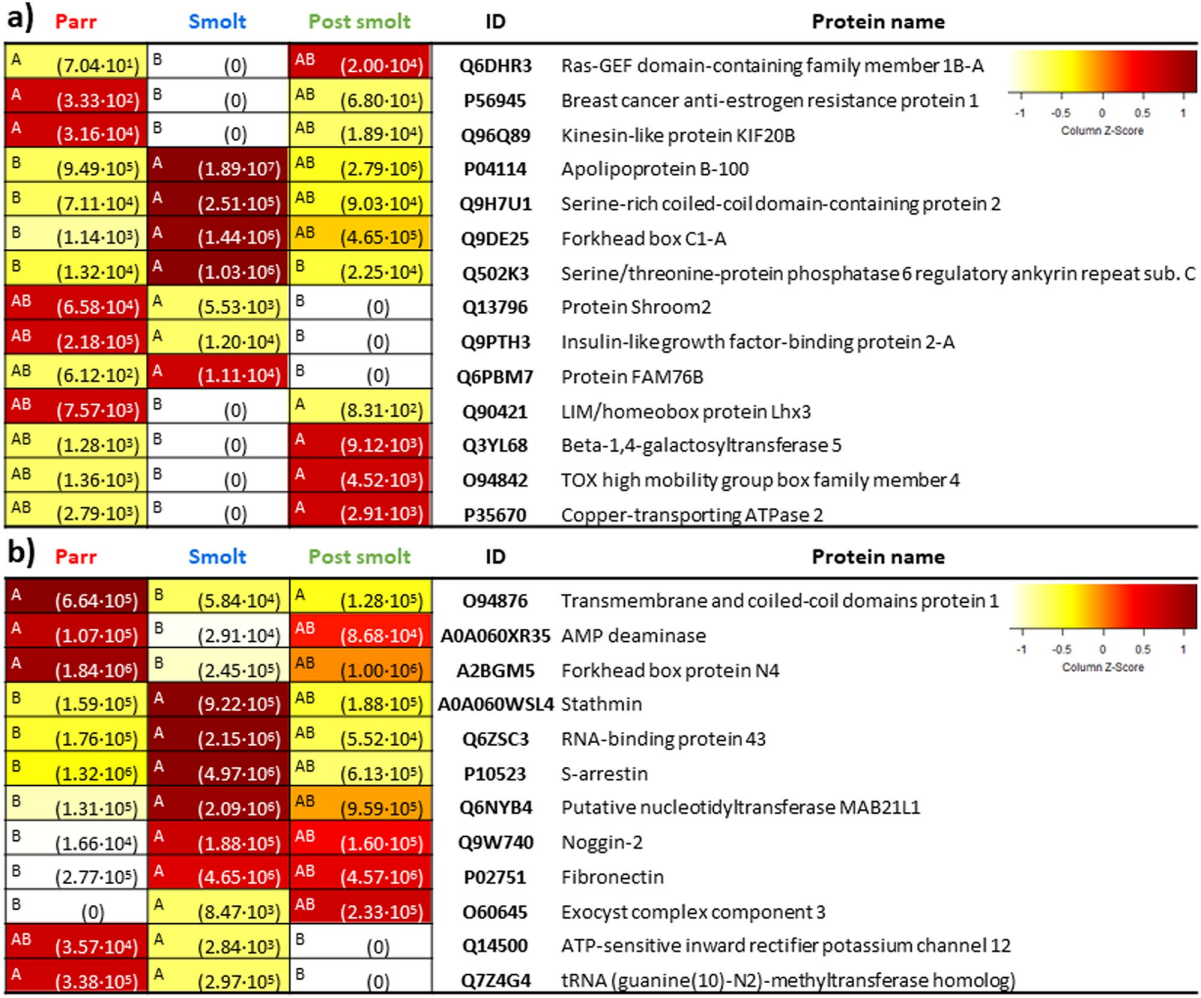
DAPs in whole (WP)(a) and enriched plasma (EP)(b). Protein abundance heatmaps indicate significant differences (ANOVA, p < 0.05, on the left of each cell different letters indicate significant differences among three plasma pool replicates per developmental condition) and mean protein abundance (in brackets in each cell). “ID” indicates UniProt accession number of the proteins. On the top right, colour scale of the heatmaps (Z-Score representing normalized ion counts).

For WP, statistical analysis revealed 7 differentially abundant proteins (q-value < 0.05, FC > 2) when comparing Parr vs. Smolt pools and 8 between the Smolt vs. Post-smolt pools comparison (Fig 6). Of these, 1 protein, Q502K3 (Serine/threonine-protein phosphatase 6 regulatory ankyrin repeat subunit), was significantly higher in the Smolt pool in both comparisons.

For EP, 10 proteins were found to be differentially abundant between the Parr vs. Smolt pools comparison, while 3 were identified for the Smolt vs. Post-smolt pools comparison (Fig 6). Of them, 1 protein, O94876 (Transmembrane and coiled-coil domains protein 1), was significantly lower in Smolt pool in both comparisons.

## 4. Discussion

Anadromous salmonids are subject to a series of adaptations needed to fulfil their unique life cycle, collectively known as smoltification [6,7]. Due to these adaptations, after having been freshwater fish during their early life stages (parr and smolt phases), anadromous salmonids can fully adapt to life in seawater during their adult stage (post-smolt phase). To gain further insight into the smoltification and seawater adaptation processes, pools of plasma from parr, smolt and post-smolt rainbow trout were analysed by GeLC-MS/MS alone or in combination with protein enrichment technology. Results were then compared seeking to identify proteins related to the changes that take place from one developmental condition to the next. Due to the integral role of blood in the transport of molecules to and from tissues, proteins from a variety of functions and target tissues were identified [40–42]. Therefore, being a highly informative biofluid, blood plasma can be used to obtain an overview of the overall physiological state of the fish [74,75].

In total, 1,822 proteins were reliably identified and quantified (based on a minimum of three peptides and one unique peptide) in rainbow trout blood plasma. Similar approaches in rainbow trout and other fish species reported a variety of protein identification results in plasma: Nynca et at. [76] reported 119 proteins in adult freshwater rainbow trout plasma (identification based on a minimum of two unique peptides). Comparison with this list of proteins revealed that 83 of them were also quantified in the present study. In zebrafish, 3,024 proteins were reported [77], 939 in three-spined sticklebacks (*Gasterosteus aculeatus*) [78], and 717 in Atlantic cod (*Gadus morhua*) (identification based on a minimum of one peptide for all three species) [79]. Interestingly, regarding other tissues and biofluids of rainbow trout, 3,241 proteins were reported in head kidney (identification based on a minimum of two peptides), 2,542 in spleen (identification based on a minimum of two peptides) [80], 59 in ovarian fluid (identification based on a minimum of two unique peptides) [81] and 152 in seminal plasma [82]. Differences in the number of protein identifications can have both biological (fish tissues and fluids used) and technical origin (sample processing, fractionation strategy, MS instrument type, data acquisition parameters, data trimming and database identification version and parameters). However, it is relevant to note that the present study represents an in-depth characterisation of the plasma proteome of rainbow trout across three developmental stages of the fish, having performed an enrichment step and extensive fractionation (each 1-D SDS-PAGE lane divided into 24 fractions), thus greatly increasing coverage.

ProteoMiner^™^ is a commercially available bead-based technology that relies on combinatorial peptide ligand libraries. Each bead is designed to specifically capture a protein up to saturation. Therefore, very abundant proteins will quickly saturate their beads and all remaining unbound protein are washed away, thus decreasing their relative abundance while increasing the concentration of low-abundant proteins [83]. Due to this, peptides that would normally be below the limit of detection of the MS or peptides that would be masked by the peptides derived from highly-abundant proteins become detectable. In the present study, WP and EP datasets were similar, with 965 proteins (52.96% of the total) being quantified by both methods and presenting very similar dynamic ranges. Nonetheless, the treatment with ProteoMiner^™^ allowed an increase in the number of quantified proteins (317 proteins, 17.95% of total). Enrichment was independent of the sample type (very similar linear regression fits between the three plasma pools), protein mass, protein length, biological function GO, molecular function GO and cellular component GO. The only tested variable significantly correlated with the magnitude of enrichment was protein abundance in the original (i.e. WP) sample, with the abundance/detectability of low-abundant proteins being greatly increased and that of high-abundant proteins decreased. This demonstrated that ProteoMiner^™^ is a valid strategy to increase proteome coverage in rainbow trout plasma. It is relevant to note that the method was more efficient at increasing the detection of low-abundant proteins than at lowering it for high-abundant ones. This was expected, as not only the relative abundance of low-abundant proteins was increased but the abundance of high-abundant proteins was reduced, which was taking up a significant portion of the available analytical space. Therefore allowing a higher proportion of the proteins present to be detected. Another effect of the enrichment was that the three developmental conditions became indistinguishable by PCA, whereas in WP they were clearly classified into three separate groups (i.e. Parr, Smolt and Post-smolt pools). Therefore, both high- and low-abundant proteins were important for the distinction of the three developmental conditions in the PCA space.

In terms of the most abundant proteins there was large variability depending on the developmental stage. However, two proteins consistently present in the top 20 of the three developmental stages were ankyrin-2 and DNA primase large subunit. In fact, ankyrin-2 was the most abundant protein in all three conditions and is a structural protein playing an essential role in the localization and retention of ion transporters and ion channels in several cell types [84]. Ankyrin-2 deficiency has been associated with blood, cardiac and neurological disorders due to its implication in the correct functioning of calcium channels and transporters. Furthermore, it is a relatively highly abundant protein in human serum [85] and its high occurrence in rainbow trout plasma is likely originating in erythrocytes, where Ankyrin-2 is crucial for correct functioning and mechanical stability [86,87]. The second most abundant common protein was DNA primase large subunit, which is a ubiquitous protein that can be found in any tissue that undergoes DNA replication. DNA primase is a polymerase that synthesizes small RNA primers for the formation of Okazaki fragments during discontinuous DNA replication [88]. Other commonly found highly abundant proteins identified in rainbow trout plasma in this study were actin, serum albumin, apolipoproteins, hemoglobin subunits, hemopexin-like proteins and complement C3, which have been reported to be high in fish plasma [89]. Although differences in the top 20 most abundant proteins were observed, only one of the proteins was significantly different among developmental stages. Thus the top abundant proteins are useful characterisers of plasma but are not critical toward understanding differences across rainbow trout life stages.

As expected from their extremely different phenotypes and life strategies, significant differences were present in the plasma proteome of Parr, Smolt and Post-smolt pools. In total, taking into consideration both WP and EP datasets, 26 DAPs were present. While some of these proteins are poorly characterised (i.e. Protein FAM76B), have associated functions that are unlikely to be found in adult fish (proteins related to embryogenesis like forkhead box protein N4 [90]) or functions that are too general (i.e. forkhead box C1-A [91]), others have well-understood and specific roles.

Seventeen DAPs were identified when comparing the Parr and Smolt pools. Their functions indicate preadaptations of smolts to seawater life, such as countermeasures against hyperosmotic stress, shown by serine/threonine-protein phosphatase 6 regulatory ankyrin repeat subunit C, a protein related the preservation of cell shape under hyperosmotic stress [92]. Retinal changes, shown by S-arrestin, a major retinal protein specific to rod photoreceptors [93–95], are likely to be linked to a signal transduction pathway related to previously documented changes in the retina of salmonids during smoltification, leading to the dominance of rhodopsin over porphyropsin as a visual pigment and to the loss of ultraviolet-sensitive cones as an adaptation to oceanic life [96,97]. Moreover, several proteins that are lower in smolts are related to a series of previously described changes related to a lower resource investment towards some nonessential processes during smoltification. One of them is sexual maturation (kinesin-like protein KIF20B [98]), which is delayed in migrators [99–101]. Another is the repression of some immune pathways [20], exemplified here by a decrease in mast cell proliferation (ras-GEF domain-containing family member 1B-A [102,103]). A third one is indicated by AMP deaminase [104] and is related to a reorganization of red muscle, resulting in a decrease in myosin heavy chain abundance and in lower performing muscles for smolts [105,106]. Parr have faster twitching muscles used to maintain a faster frequency tailbeat, whereas smolts tend to swim with the current towards the river mouth. Finally, changes in energy reserve metabolism result in depletion of lipids, proteins and carbohydrates in the smolt whole body [107], which is shown here by a higher mobilization of lipids (apolipoprotein B-100 [108,109]). Presumably these adaptations would take place in order to meet the energy demands needed for smoltification and for reorganizing tissues in preparation to life in seawater, which could be related to the higher abundance in smolts of serine-rich coiled-coil domain-containing protein 2, stathmin and fibronectin, all related to cell and microtubule organization [110–112]. Curiously, serine-rich coiled-coil domain-containing protein 2 has been proposed as a reliable housekeeping gene in humans but this is unlikely to be the case in rainbow trout given the present results [113].

For the Smolt vs Post-smolt pools comparison, 11 DAPs were identified. They were related to countermeasures against hyperosmotic stress, shown once again by the high abundance of serine/threonine-protein phosphatase 6 regulatory ankyrin repeat subunit C, and by ATP-sensitive inward rectifier potassium channel 12, a channel with the tendency to let potassium into cells [114], being likely related to the seawater NKA pumps of rainbow trout (NKAα1b and NKCC1a). Finally, IGFBP2a was absent in Post-smolt. The absence of this growth-repressing protein indicates that growth during the post-smolt phase (also called on-growing phase) would be enhanced with respect to the smolt phase [115,116], which is in accordance with the repression of nonessential processes during smoltification as mentioned above.

Overall, the present study provides an in-depth characterisation of the rainbow trout blood plasma proteome, with 1,822 reliably identified and quantified proteins, analysed across three different developmental stages of the fish. Performing either fractionation alone or in combination with an enrichment step effectively maximized proteome coverage, supporting previous findings in human proteomics [117]. The effects of ProteoMiner^™^ were explored in-depth, showing that this can increase the number of detected proteins due to the capability to decrease the masking effect on high-abundant proteins. In general terms, ankyrin-2 was invariably the most abundant protein in rainbow trout plasma, while other proteins such as DNA primase large subunit, actin, serum albumin, apolipoproteins, hemoglobin subunits, hemopexin-like proteins and complement C3 were generally among the most abundant proteins. DAPs between Parr and Smolt pools suggest preadaptations of smolts to seawater life, including prevention measures against hyperosmotic stress and retinal changes and mobilization of energetic resources, as well as downregulation in respect to the Parr pool of nonessential pathways. DAPs between Smolt and Post-smolt pools were related to coping with hyperosmotic stress, retinal changes, and increased growth and copper excretion in Post-smolt pool. As potential biomarkers, apolipoprotein B-100 would be the clearest candidate, being a highly abundant protein (second most abundant in Smolt pool) and significantly higher in Smolt pool than in Parr or Post-smolt pools. Therefore, it might be a robust smolt marker, increasing during the smoltification process and decreasing after the smolt window. However, being and important lipoprotein component, it is implicated in the transport of lipids in response to a wide variety of signals [118–120] and its abundance might vary in response to processes not related to smoltification. Therefore, its suitability as smolt marker, as for the rest of potential biomarkers, needs further testing. For post-smolts, the lack of IGFBP2a might also be an interesting biomarker for growth potential. Other biomarker candidates could preferably be DAPs found in WP, due to the simplicity of detection respect to EP. However, each of these candidate biomarkers needs to be validated by seawater survival tests.

## Supporting information

S1 Fig Detected unique peptides. Proteins for which 1 (a), 2 (b), 3 (c), 4 (d), 5 (e), or more than 5 (f) unique peptides were detected. Proteins are arranged according to their dynamic range. Red points indicate proteins that meet the criteria of the corresponding panel.
(TIF)

S2 Fig Number of unique proteins and protein mass and length. Correlation between the number of unique proteins with protein mass (a,c) and amino acid length (b,d) in whole (WP)(a,b) and in enriched plasma (EP)(c, d).
(TIF)

S3 Fig One-dimensional SDS PAGE of Post-smolt and parr whole (WP) and enriched plasma (EP). Gel image (a), densitometry of Post-smolt pool WP and EP (b) and densitometry of Parr pool WP and EP (c). Dashed line indicates where gel was edited to remove unwanted gel lanes. Original gel image is provided as S1 Raw images.
(TIF)

S4 Fig Gene ontology (GO) terms associated to proteins quantified in whole plasma and enriched plasma. Biological GO (a), cellular component GO (b) and molecular function GO (c).
(TIF)

S1 File Peptides detected in whole plasma (WP). (XLSX)

S2 File Proteins detected in whole plasma (WP). (XLSX)

S3 File Peptides detected in enriched plasma (EP). (XLSX)

S4 File Proteins detected in enriched plasma (EP). (XLSX)

S1 Raw images (TIF)

## References

[1] Fleming IA, Reynolds JD. Salmonid breeding systems. Evolution Illuminated: Salmon and Their Relatives. 2004: 264–294.

[2] Quinn TP, Seamons TR, Vollestad LA, Duffy E. Effects of growth and reproductive history on the egg size-fecundity trade-off in steelhead. Trans Am Fish Soc. 2011;140: 45–51.

[3] Quinn TP, McGinnity P, Reed TE. The paradox of “premature migration” by adult anadromous salmonid fishes: Patterns and hypotheses. Can J Fish Aquatic Sci. 2016;73: 1015–1030.

[4] Kendall NW, McMillan JR, Sloat MR, Buehrens TW, Quinn TP, Pess GR, et al Anadromy and residency in steelhead and rainbow trout (*Oncorhynchus mykiss*): A review of the Processes and Patterns. Can J Fish Aquatic Sci. 2015;72: 319–342.

[5] Prunet P, Boeuf G, Bolton JP, Young G. Smoltification and seawater adaptation in Atlantic salmon (*Salmo salar*): Plasma prolactin, growth hormone, and thyroid hormones. Gen Comp Endocrinol. 1989;74: 355–364. 10.1016/s0016-6480(89)80031-0 2545514

[6] Hoar WS. The physiology of smolting salmonids. Fish Physiology. 1988;11 B: 275–343.

[7] Björnsson BT, Stefansson SO, McCormick SD. Environmental endocrinology of salmon smoltification. Gen Comp Endocrinol. 2011;170: 290–298. 10.1016/j.ygcen.2010.07.003 20627104

[8] Mancera JM, McCormick SD. Role of prolactin, growth hormone, insulin-like growth factor and cortisol in teleost osmoregulation. Fish Osmoregulation. 2007: 497–515.

[9] McCormick SD. Endocrine control of osmoregulation in teleost fish. Am Zool. 2001;41: 781–794.

[10] Winans GA, Nishioka RS. A multivariate description of change in body shape of coho salmon (*Oncorhynchus kisutch*) during smoltification. Aquaculture. 1987;66: 235–245.

[11] Riley WD, Ibbotson AT, Maxwell DL, Davison PI, Beaumont WRC, Ives MJ. Development of schooling behaviour during the downstream migration of Atlantic salmon *Salmo salar* smolts in a chalk stream. J Fish Biol. 2014;85: 1042–1059. 10.1111/jfb.12457 25052817

[12] Fontaine M, Hatey J. Variations of the liver glycogen in the young (*Salmo salar* L.) during smoltification. C R Seances Soc Biol Fil. 1950;144: 953–955. 14792810

[13] Kobayashi R, yuki R. Differences in Catalase Activity in the Tissues and Blood between the Smolt and Parr of Masu, *Oncorhynchus masou*. Bulletin of The Faculty of Fisheries Hokkaido University. 1954;5: 223–230.

[14] Ebbesson LOE, Björnsson BT, Ekström P, Stefansson SO. Daily endocrine profiles in parr and smolt Atlantic salmon. Comp Biochem Physiol A Mol Integr Physiol. 2008;151: 698–704. 10.1016/j.cbpa.2008.08.017 18790069

[15] Handeland SO, Imsland AK, Björnsson BT, Stefansson SO. Long-term effects of photoperiod, temperature and their interaction on growth, gill Na+, K+-ATPase activity, seawater tolerance and plasma growth-hormone levels in Atlantic salmon *Salmo salar*. J Fish Biol. 2013;83: 1197–1209. 10.1111/jfb.12215 24580662

[16] McCormick SD, Regish AM, Christensen AK. Distinct freshwater and seawater isoforms of Na^+^/K^+^-ATPase in gill chloride cells of Atlantic salmon. J Exp Biol. 2009;212: 3994–4001. 10.1242/jeb.037275 19946077

[17] Zaugg WS, Wagner HH. Gill ATPase activity related to parr-smolt transformation and migration in steelhead trout (*Salmo gairdneri*): Influence of photoperiod and temperature. Comparative Biochemistry and Physiology—Part B: Biochemistry and. 1973;45: 955–965. 10.1016/0305-0491(73)90156-9 4269550

[18] Berthelot C, Brunet F, Chalopin D, Juanchich A, Bernard M, Noel B, et al The rainbow trout genome provides novel insights into evolution after whole-genome duplication in vertebrates. Nat Commun. 2014;5: 3657 10.1038/ncomms4657 24755649 PMC4071752

[19] Davidson WS, Koop BF, Jones SJM, Iturra P, Vidal R, Maass A, et al Sequencing the genome of the Atlantic salmon (*Salmo salar*). Genome Biol. 2010;11. 10.1186/gb-2010-11-9-403 PMC2965382 20887641

[20] Johansson L, Timmerhaus G, Afanasyev S, Jørgensen SM, Krasnov A. Smoltification and seawater transfer of Atlantic salmon (*Salmo salar* L.) is associated with systemic repression of the immune transcriptome. Fish Shellfish Immunol. 2016;58: 33–41. 10.1016/j.fsi.2016.09.026 27637733

[21] Norman JD, Ferguson MM, Danzmann RG. An integrated transcriptomic and comparative genomic analysis of differential gene expression in Arctic charr (*Salvelinus alpinus*) following seawater exposure. J Exp Biol. 2014;217: 4029–4042. 10.1242/jeb.107441 25278466

[22] Norman JD, Ferguson MM, Danzmann RG. Transcriptomics of salinity tolerance capacity in Arctic charr (*Salvelinus alpinus*): a comparison of gene expression profiles between divergent QTL genotypes. American Journal of Physiology-Heart and Circulatory Physiology. 2013. 10.1152/physiolgenomics.00105.2013 PMC3921346 24368751

[23] Baerwald MR, Meek MH, Stephens MR, Nagarajan RP, Goodbla AM, Tomalty KMH, et al Migration-related phenotypic divergence is associated with epigenetic modifications in rainbow trout. Mol Ecol. 2016;25: 1785–1800. 10.1111/mec.13231 25958780 PMC4641842

[24] Hecht BC, Thrower FP, Hale MC, Miller MR, Nichols KM. Genetic architecture of migration-related traits in rainbow and steelhead trout, *Oncorhynchus mykiss*. G3 (Bethesda). 2012;2: 1113–1127. 22973549 10.1534/g3.112.003137 PMC3429926

[25] Hecht BC, Valle ME, Thrower FP, Nichols KM. Divergence in expression of candidate genes for the smoltification process between juvenile resident rainbow and anadromous steelhead trout. Mar Biotechnol (NY). 2014;16: 638–656. 24952010 10.1007/s10126-014-9579-7

[26] Sutherland BJ, Hanson KC, Jantzen JR, Koop BF, Smith CT. Divergent immunity and energetic programs in the gills of migratory and resident *Oncorhynchus mykiss*. Mol Ecol. 2014;23: 1952–1964. 10.1111/mec.12713 24612010

[27] Hale MC, McKinney GJ, Thrower FP, Nichols KM. RNA-seq reveals differential gene expression in the brains of juvenile resident and migratory smolt rainbow trout (*Oncorhynchus mykiss*). Comparative Biochemistry and Physiology Part D: Genomics and Proteomics. 2016;20: 136–150. 10.1016/j.cbd.2016.07.006 27693967

[28] Shimomura T, Nakajima T, Horikoshi M, Iijima A, Urabe H, Mizuno S, et al Relationships between gill Na+,K+-ATPase activity and endocrine and local insulin-like growth factor-I levels during smoltification of masu salmon (*Oncorhynchus masou*). Gen Comp Endocrinol. 2012;178: 427–435. 10.1016/j.ygcen.2012.06.011 22749841

[29] Beckman BR, Shimizu M, Gadberry BA, Cooper KA. Response of the somatotropic axis of juvenile coho salmon to alterations in plane of nutrition with an analysis of the relationships among growth rate and circulating IGF-I and 41 kDa IGFBP. Gen Comp Endocrinol. 2004;135: 334–344. 10.1016/j.ygcen.2003.10.013 14723885

[30] Björnsson BT, Bradley TM. Epilogue: Past successes, present misconceptions and future milestones in salmon smoltification research. Aquaculture. 2007;273: 384–391.

[31] Zhu W, Smith JW, Huang CM. Mass spectrometry-based label-free quantitative proteomics. J Biomed Biotechnol. 2010;2010: 840518 10.1155/2010/840518 19911078 PMC2775274

[32] Geromanos SJ, Vissers JP, Silva JC, Dorschel CA, Li G, Gorenstein MV, et al The detection, correlation, and comparison of peptide precursor and product ions from data independent LC-MS with data dependant LC-MS/MS. Proteomics. 2009;9: 1683–1695. 10.1002/pmic.200800562 19294628

[33] Liumbruno G, D’Alessandro A, Grazzini G, Zolla L. Blood-related proteomics. Journal of proteomics. 2010;73: 483–507. 10.1016/j.jprot.2009.06.010 19567275

[34] Barnea E, Sorkin R, Ziv T, Beer I, Admon A. Evaluation of prefractionation methods as a preparatory step for multidimensional based chromatography of serum proteins. Proteomics. 2005;5: 3367–3375. 10.1002/pmic.200401221 16047308

[35] Fang Y, Gao X, Zha J, Ning B, Li X, Gao Z, et al Identification of differential hepatic proteins in rare minnow (*Gobiocypris rarus*) exposed to pentachlorophenol (PCP) by proteomic analysis. Toxicol Lett. 2010;199: 69–79. 10.1016/j.toxlet.2010.08.008 20732397

[36] Corthals GL, Wasinger VC, Hochstrasser DF, Sanchez J. The dynamic range of protein expression: a challenge for proteomic research. Electrophoresis: An International Journal. 2000;21: 1104–1115. 10.1002/(SICI)1522-2683(20000401)21:6<1104::AID-ELPS1104>3.0.CO;2-C 10786884

[37] Bandow JE. Comparison of protein enrichment strategies for proteome analysis of plasma. Proteomics. 2010;10: 1416–1425. 10.1002/pmic.200900431 20127685

[38] Millioni R, Tolin S, Puricelli L, Sbrignadello S, Fadini GP, Tessari P, et al High abundance proteins depletion vs low abundance proteins enrichment: comparison of methods to reduce the plasma proteome complexity. PloS one. 2011;6: e19603 10.1371/journal.pone.0019603 21573190 PMC3087803

[39] De Bock M, De Seny D, Meuwis M, Servais A, Minh TQ, Closset J, et al Comparison of three methods for fractionation and enrichment of low molecular weight proteins for SELDI-TOF-MS differential analysis. Talanta. 2010;82: 245–254. 10.1016/j.talanta.2010.04.029 20685463

[40] Jacobs JM, Adkins JN, Qian W, Liu T, Shen Y, Camp DG, et al Utilizing human blood plasma for proteomic biomarker discovery. Journal of proteome research. 2005;4: 1073–1085. 10.1021/pr0500657 16083256

[41] Anderson NL, Anderson NG. The human plasma proteome: history, character, and diagnostic prospects. Mol Cell Proteomics. 2002;1: 845–867. 10.1074/mcp.r200007-mcp200 12488461

[42] Pernemalm M, Lehtiö J. Mass spectrometry-based plasma proteomics: state of the art and future outlook. Expert review of proteomics. 2014;11: 431–448. 10.1586/14789450.2014.901157 24661227

[43] Geyer PE, Holdt LM, Teupser D, Mann M. Revisiting biomarker discovery by plasma proteomics. Mol Syst Biol. 2017;13: 942 10.15252/msb.20156297 28951502 PMC5615924

[44] Hanash SM, Pitteri SJ, Faca VM. Mining the plasma proteome for cancer biomarkers. Nature. 2008;452: 571 10.1038/nature06916 18385731

[45] Hye A, Lynham S, Thambisetty M, Causevic M, Campbell J, Byers H, et al Proteome-based plasma biomarkers for Alzheimer’s disease. Brain. 2006;129: 3042–3050. 10.1093/brain/awl279 17071923

[46] Beckman BR. Perspectives on concordant and discordant relations between insulin-like growth factor 1 (IGF1) and growth in fishes. Gen Comp Endocrinol. 2011;170: 233–252. 10.1016/j.ygcen.2010.08.009 20800595

[47] Beckman BR, Fairgrieve W, Cooper KA, Mahnken CVW, Beamish RJ. Evaluation of endocrine indices of growth in individual postsmolt coho salmon. Trans Am Fish Soc. 2004;133: 1057–1067.

[48] O’Loughlin A, McGee M, Doyle S, Earley B. Biomarker responses to weaning stress in beef calves. Res Vet Sci. 2014;97: 458–463. 10.1016/j.rvsc.2014.06.003 24992823

[49] Fast MD, Hosoya S, Johnson SC, Afonso LO. Cortisol response and immune-related effects of Atlantic salmon (*Salmo salar* Linnaeus) subjected to short-and long-term stress. Fish Shellfish Immunol. 2008;24: 194–204. 10.1016/j.fsi.2007.10.009 18065240

[50] Palermo F, Mosconi G, Angeletti M, Polzonetti-Magni A. Assessment of water pollution in the Tronto River (Italy) by applying useful biomarkers in the fish model *Carassius auratus*. Arch Environ Contam Toxicol. 2008;55: 295–304. 10.1007/s00244-007-9113-2 18214578

[51] Hiramatsu N, Matsubara T, Fujita T, Sullivan CV, Hara A. Multiple piscine vitellogenins: biomarkers of fish exposure to estrogenic endocrine disruptors in aquatic environments. Mar Biol. 2006;149: 35–47.

[52] Barton C, Beck P, Kay R, Teale P, Roberts J. Multiplexed LC-MS/MS analysis of horse plasma proteins to study doping in sport. Proteomics. 2009;9: 3058–3065. 10.1002/pmic.200800737 19526555

[53] Morro B, Balseiro P, Albalat A, Pedrosa C, MacKenzie S, Nakamura S, et al Effects of different photoperiod regimes on the smoltification and seawater adaptation of seawater-farmed rainbow trout (*Oncorhynchus mykiss*): Insights from Na^+^, K^+^–ATPase activity and transcription of osmoregulation and growth regulation genes. Aquaculture. 2019;507: 282–292.

[54] McCormick SD. Methods for nonlethal gill biopsy and measurement of Na^+^,K^+^-ATPase activity. Can J Fish Aquat Sci. 1993;50: 656–658.

[55] Madsen S, Naamansen E. Plasma ionic regulation and gill Na /K -ATPase changes during rapid transfer to sea water of yearling rainbow trout, *Salmo gairdneri*: time course and seasonal variation. J Fish Biol. 1989;34: 829–840.

[56] Ewing R, Barratt D, Garlock D. Physiological changes related to migration tendency in rainbow trout (*Oncorhynchus mykiss*). Aquaculture. 1994;121: 277–287.

[57] Zhang W, Carriquiry A, Nettleton D, Dekkers JC. Pooling mRNA in microarray experiments and its effect on power. Bioinformatics. 2007;23: 1217–1224. 10.1093/bioinformatics/btm081 17344238

[58] Weinkauf M, Hiddemann W, Dreyling M. Sample pooling in 2-D gel electrophoresis: A new approach to reduce nonspecific expression background. Electrophoresis. 2006;27: 4555–4558. 10.1002/elps.200600207 17066383

[59] Neubauer H, Clare SE, Kurek R, Fehm T, Wallwiener D, Sotlar K, et al Breast cancer proteomics by laser capture microdissection, sample pooling, 54-cm IPG IEF, and differential iodine radioisotope detection. Electrophoresis. 2006;27: 1840–1852. 10.1002/elps.200500739 16645950

[60] Horgan GW. Sample size and replication in 2D gel electrophoresis studies. Journal of proteome research. 2007;6: 2884–2887. 10.1021/pr070114a 17550277

[61] Karp NA, Lilley KS. Design and analysis issues in quantitative proteomics studies. Proteomics. 2007;7: 42–50. 10.1002/pmic.200700683 17893850

[62] Karp NA, Spencer M, Lindsay H, O’Dell K, Lilley KS. Impact of replicate types on proteomic expression analysis. Journal of proteome research. 2005;4: 1867–1871. 10.1021/pr050084g 16212444

[63] Diz AP, Truebano M, Skibinski DO. The consequences of sample pooling in proteomics: an empirical study. Electrophoresis. 2009;30: 2967–2975. 10.1002/elps.200900210 19676090

[64] Zolg W. The proteomic search for diagnostic biomarkers: Lost in translation? Mol Cell Proteomics. 2006;5: 1720–1726. 10.1074/mcp.R600001-MCP200 16546995

[65] Karp NA, Lilley KS. Investigating sample pooling strategies for DIGE experiments to address biological variability. Proteomics. 2009;9: 388–397. 10.1002/pmic.200800485 19105178

[66] Shih JH, Michalowska AM, Dobbin K, Ye Y, Qiu TH, Green JE. Effects of pooling mRNA in microarray class comparisons. Bioinformatics. 2004;20: 3318–3325. 10.1093/bioinformatics/bth391 15247103

[67] Martínez-Fernández M, Rodríguez-Piñeiro AM, Oliveira E, Páez de la Cadena María, Rolán-Alvarez E. Proteomic comparison between two marine snail ecotypes reveals details about the biochemistry of adaptation. Journal of proteome research. 2008;7: 4926–4934. 10.1021/pr700863e 18937509

[68] Kendziorski C, Irizarry RA, Chen KS, Haag JD, Gould MN. On the utility of pooling biological samples in microarray experiments. Proc Natl Acad Sci U S A. 2005;102: 4252–4257. 10.1073/pnas.0500607102 15755808 PMC552978

[69] Paulo JA. Practical and Efficient Searching in Proteomics: A Cross Engine Comparison. Webmedcentral. 2013;4: 10.9754/journal.wplus.2013.0052 25346847 PMC4208621

[70] Wickham H. ggplot2 Elegant Graphics for Data Analysis Introduction; 2009.

[71] Li G, Vissers JP, Silva JC, Golick D, Gorenstein MV, Geromanos SJ. Database searching and accounting of multiplexed precursor and product ion spectra from the data independent analysis of simple and complex peptide mixtures. Proteomics. 2009;9: 1696–1719. 10.1002/pmic.200800564 19294629

[72] Bhatia VN, Perlman DH, Costello CE, McComb ME. Software tool for researching annotations of proteins: open-source protein annotation software with data visualization. Anal Chem. 2009;81: 9819–9823. 10.1021/ac901335x 19839595 PMC2787672

[73] Vu VQ. ggbiplot: A ggplot2 based biplot. R package. 2011;342.

[74] Adkins JN, Monroe ME, Auberry KJ, Shen Y, Jacobs JM, Camp DG II, et al A proteomic study of the HUPO Plasma Proteome Project’s pilot samples using an accurate mass and time tag strategy. Proteomics. 2005;5: 3454–3466. 10.1002/pmic.200401333 16052625 PMC2041806

[75] Simpson KL, Whetton AD, Dive C. Quantitative mass spectrometry-based techniques for clinical use: biomarker identification and quantification. Journal of Chromatography B. 2009;877: 1240–1249. 10.1016/j.jchromb.2008.11.023 PMC7185464 19058768

[76] Nynca J, Arnold G, Fröhlich T, Ciereszko A. Proteomic identification of rainbow trout blood plasma proteins and their relationship to seminal plasma proteins. Proteomics. 2017;17: 1600460. 10.1002/pmic.201600460 28394474

[77] Medina-Gali R, Belló-Pérez M, Ciordia S, Mena MC, Coll J, Novoa B, et al Plasma proteomic analysis of zebrafish following spring viremia of carp virus infection. Fish Shellfish Immunol. 2019;86: 892–899. 10.1016/j.fsi.2018.12.035 30580041

[78] Kültz D, Li J, Zhang X, Villarreal F, Pham T, Paguio D. Population-specific plasma proteomes of marine and freshwater three-spined sticklebacks (*Gasterosteus aculeatus*). Proteomics. 2015;15: 3980–3992. 10.1002/pmic.201500132 26223892

[79] Enerstvedt KS, Sydnes MO, Pampanin DM. Study of the plasma proteome of Atlantic cod (*Gadus morhua*): Changes due to crude oil exposure. Mar Environ Res. 2018;138: 46–54. 10.1016/j.marenvres.2018.03.009 29692335

[80] Kumar G, Hummel K, Razzazi-Fazeli E, El-Matbouli M. Proteome Profiles of Head Kidney and Spleen of Rainbow Trout (*Oncorhynchus mykiss*). Proteomics. 2018;18: 1800101. 10.1002/pmic.201800101 PMC6175351 30094954

[81] Nynca J, Arnold GJ, Fröhlich T, Ciereszko A. Shotgun proteomics of rainbow trout ovarian fluid. Reproduction, Fertility and Development. 2015;27: 504–512. 10.1071/RD13224 25482144

[82] Nynca J, Arnold GJ, Fröhlich T, Otte K, Flenkenthaler F, Ciereszko A. Proteomic identification of rainbow trout seminal plasma proteins. Proteomics. 2014;14: 133–140. 10.1002/pmic.201300267 24174285

[83] Murphy S, Dowling P. DIGE Analysis of ProteoMiner TM Fractionated Serum/Plasma Samples In: Anonymous Difference Gel Electrophoresis. Springer; 2018 pp. 109–114.

[84] Cunha SR, Mohler PJ. Ankyrin protein networks in membrane formation and stabilization. J Cell Mol Med. 2009;13: 4364–4376. 10.1111/j.1582-4934.2009.00943.x 19840192 PMC4515052

[85] Tanaka Y, Akiyama H, Kuroda T, Jung G, Tanahashi K, Sugaya H, et al A novel approach and protocol for discovering extremely low-abundance proteins in serum. Proteomics. 2006;6: 4845–4855. 10.1002/pmic.200500774 16878292

[86] Mohandas N, Evans E. Mechanical properties of the red cell membrane in relation to molecular structure and genetic defects. Annu Rev Biophys Biomol Struct. 1994;23: 787–818. 10.1146/annurev.bb.23.060194.004035 7919799

[87] Yasunaga M, Ipsaro JJ, Mondragón A. Structurally similar but functionally diverse ZU5 domains in human erythrocyte ankyrin. J Mol Biol. 2012;417: 336–350. 10.1016/j.jmb.2012.01.041 22310050 PMC3312341

[88] Kuchta RD, Stengel G. Mechanism and evolution of DNA primases. Biochimica et Biophysica Acta (BBA)-Proteins and Proteomics. 2010;1804: 1180–1189. 19540940 10.1016/j.bbapap.2009.06.011 PMC2846230

[89] Li C, Tan XF, Lim TK, Lin Q, Gong Z. Comprehensive and quantitative proteomic analyses of zebrafish plasma reveals conserved protein profiles between genders and between zebrafish and human. Scientific reports. 2016;6: 24329 10.1038/srep24329 27071722 PMC4829857

[90] Chi NC, Shaw RM, De Val S, Kang G, Jan LY, Black BL, et al Foxn4 directly regulates tbx2b expression and atrioventricular canal formation. Genes Dev. 2008;22: 734–739. 10.1101/gad.1629408 18347092 PMC2275426

[91] Li J, Yue Y, Dong X, Jia W, Li K, Liang D, et al Zebrafish foxc1a plays a crucial role in early somitogenesis by restricting the expression of aldh1a2 directly. J Biol Chem. 2015;290: 10216–10228. 10.1074/jbc.M114.612572 25724646 PMC4400337

[92] Watanabe K, Umeda T, Niwa K, Naguro I, Ichijo H. A PP6-ASK3 module coordinates the bidirectional cell volume regulation under osmotic stress. Cell reports. 2018;22: 2809–2817. 10.1016/j.celrep.2018.02.045 29539411

[93] Sullivan LS, Bowne SJ, Koboldt DC, Cadena EL, Heckenlively JR, Branham KE, et al A novel dominant mutation in SAG, the arrestin-1 gene, is a common cause of retinitis pigmentosa in Hispanic families in the Southwestern United States. Invest Ophthalmol Vis Sci. 2017;58: 2774–2784. 10.1167/iovs.16-21341 28549094 PMC5455168

[94] Renninger SL, Gesemann M, Neuhauss SC. Cone arrestin confers cone vision of high temporal resolution in zebrafish larvae. Eur J Neurosci. 2011;33: 658–667. 10.1111/j.1460-9568.2010.07574.x 21299656

[95] Murthy KR, Goel R, Subbannayya Y, Jacob HK, Murthy PR, Manda SS, et al Proteomic analysis of human vitreous humor. Clinical proteomics. 2014;11: 29 10.1186/1559-0275-11-29 25097467 PMC4106660

[96] Dann SG, Allison WT, Levin DB, Hawryshyn CW. Identification of a unique transcript down-regulated in the retina of rainbow trout (*Oncorhynchus mykiss*) at smoltification. Comp Biochem Physiol B Biochem Mol Biol. 2003;136: 849–860. 10.1016/s1096-4959(03)00262-8 14662307

[97] Ebbesson LO, Ebbesson SO, Nilsen TO, Stefansson SO, Holmqvist B. Exposure to continuous light disrupts retinal innervation of the preoptic nucleus during parr–smolt transformation in Atlantic salmon. Aquaculture. 2007;273: 345–349.

[98] Wang X, Liu Q, Xu S, Xiao Y, Wang Y, Feng C, et al Transcriptome Dynamics During Turbot Spermatogenesis Predicting the Potential Key Genes Regulating Male Germ Cell Proliferation and Maturation. Scientific reports. 2018;8: 15825 10.1038/s41598-018-34149-5 30361543 PMC6202422

[99] Foote CJ, Mayer I, Wood CC, Clarke WC, Blackburn J. On the developmental pathway to nonanadromy in sockeye salmon, Oncorhynchus nerka. Can J Zool. 1994;72: 397–405.

[100] Thorpe JE, Metcalfe NB. Is smolting a positive or a negative developmental decision? Aquaculture. 1998;168: 95–103.

[101] Nichols KM, Edo AF, Wheeler PA, Thorgaard GH. The genetic basis of smoltification-related traits in *Oncorhynchus mykiss*. Genetics. 2008;179: 1559–1575. 10.1534/genetics.107.084251 18562654 PMC2475755

[102] Tam S, Tsai M, Snouwaert JN, Kalesnikoff J, Scherrer D, Nakae S, et al RabGEF1 is a negative regulator of mast cell activation and skin inflammation. Nat Immunol. 2004;5: 844 10.1038/ni1093 15235600

[103] Epting D, Vorwerk S, Hageman A, Meyer D. Expression of rasgef1b in zebrafish. Gene expression patterns. 2007;7: 389–395. 10.1016/j.modgep.2006.11.010 17239665

[104] Fischer H, Esbjörnsson M, Sabina RL, Strömberg A, Peyrard-Janvid M, Norman B. AMP deaminase deficiency is associated with lower sprint cycling performance in healthy subjects. J Appl Physiol. 2007. 10.1152/japplphysiol.00185.2007 17463303

[105] Coughlin DJ, Forry JA, McGlinchey SM, Mitchell J, Saporetti KA, Stauffer KA. Thyroxine induces transitions in red muscle kinetics and steady swimming kinematics in rainbow trout (*Oncorhynchus mykiss*). J Exp Zool. 2001;290: 115–124. 10.1002/jez.1041 11471141

[106] Martinez I, Bang B, Hatlen B, Blix P. Myofibrillar proteins in skeletal muscles of parr, smolt and adult atlantic salmon (*Salmo salar* l.). Comparison with another salmonid, the arctic charr *Salvelinus alpinus* (l.). Comparative Biochemistry and Physiology—Part B: Biochemistry and. 1993;106: 1021–1028.

[107] Rousseau K, Martin P, Boeuf G, Dufour S. Salmonid smoltification Metamorphosis in fish. CRC Press, Boca Raton 2012: 167–215.

[108] Millar JS, Maugeais C, Ikewaki K, Kolansky DM, Barrett PHR, Budreck EC, et al Complete deficiency of the low-density lipoprotein receptor is associated with increased apolipoprotein B-100 production. Arterioscler Thromb Vasc Biol. 2005;25: 560–565. 10.1161/01.ATV.0000155323.18856.a2 15637307

[109] Innerarity TL, Mahley RW, Weisgraber KH, Bersot TP, Krauss RM, Vega GL, et al Familial defective apolipoprotein B-100: a mutation of apolipoprotein B that causes hypercholesterolemia. J Lipid Res. 1990;31: 1337–1349. 2280177

[110] Pankov R, Yamada KM. Fibronectin at a glance. J Cell Sci. 2002;115: 3861–3863. 10.1242/jcs.00059 12244123

[111] Wang J, Karra R, Dickson AL, Poss KD. Fibronectin is deposited by injury-activated epicardial cells and is necessary for zebrafish heart regeneration. Dev Biol. 2013;382: 427–435. 10.1016/j.ydbio.2013.08.012 23988577 PMC3852765

[112] Rubin CI, Atweh GF. The role of stathmin in the regulation of the cell cycle. J Cell Biochem. 2004;93: 242–250. 10.1002/jcb.20187 15368352

[113] Tilli TM, da Silva Castro C, Tuszynski JA, Carels N. A strategy to identify housekeeping genes suitable for analysis in breast cancer diseases. BMC Genomics. 2016;17: 639 10.1186/s12864-016-2946-1 27526934 PMC4986254

[114] D’Avanzo N, Cheng WW, Doyle DA, Nichols CG. Direct and specific activation of human inward rectifier K^+^ channels by membrane phosphatidylinositol 4,5-bisphosphate. J Biol Chem. 2010;285: 37129–37132. 10.1074/jbc.C110.186692 20921230 PMC2988318

[115] Zhou J, Li W, Kamei H, Duan C. Duplication of the IGFBP-2 gene in teleost fish: protein structure and functionality conservation and gene expression divergence. PloS one. 2008;3: e3926 10.1371/journal.pone.0003926 19081843 PMC2593780

[116] Duan C, Ding J, Li Q, Tsai W, Pozios K. Insulin-like growth factor binding protein 2 is a growth inhibitory protein conserved in zebrafish. Proc Natl Acad Sci U S A. 1999;96: 15274–15279. 10.1073/pnas.96.26.15274 10611375 PMC24810

[117] Selvaraju S, El Rassi Z. Reduction of protein concentration range difference followed by multicolumn fractionation prior to 2-DE and LC-MS/MS profiling of serum proteins. Electrophoresis. 2011;32: 674–685. 10.1002/elps.201000606 21365658

[118] Klingenberg R, Lebens M, Hermansson A, Fredrikson GN, Strodthoff D, Rudling M, et al Intranasal immunization with an apolipoprotein B-100 fusion protein induces antigen-specific regulatory T cells and reduces atherosclerosis. Arterioscler Thromb Vasc Biol. 2010;30: 946–952. 10.1161/ATVBAHA.109.202671 20167655

[119] Kreuter J, Hekmatara T, Dreis S, Vogel T, Gelperina S, Langer K. Covalent attachment of apolipoprotein AI and apolipoprotein B-100 to albumin nanoparticles enables drug transport into the brain. J Controlled Release. 2007;118: 54–58. 10.1016/j.jconrel.2006.12.012 17250920

[120] Segrest JP, Jones MK, De Loof H, Dashti N. Structure of apolipoprotein B-100 in low density lipoproteins. J Lipid Res. 2001;42: 1346–1367. 11518754

