# Supplementary figures and images for: Plasma proteome profiling of freshwater and seawater life stages of rainbow trout (Oncorhynchus mykiss)
Source: PLoS One. 2020 Jan 3;15(1):e0227003. doi: 10.1371/journal.pone.0227003 (PMC6941806; doi:10.1371/journal.pone.0227003)

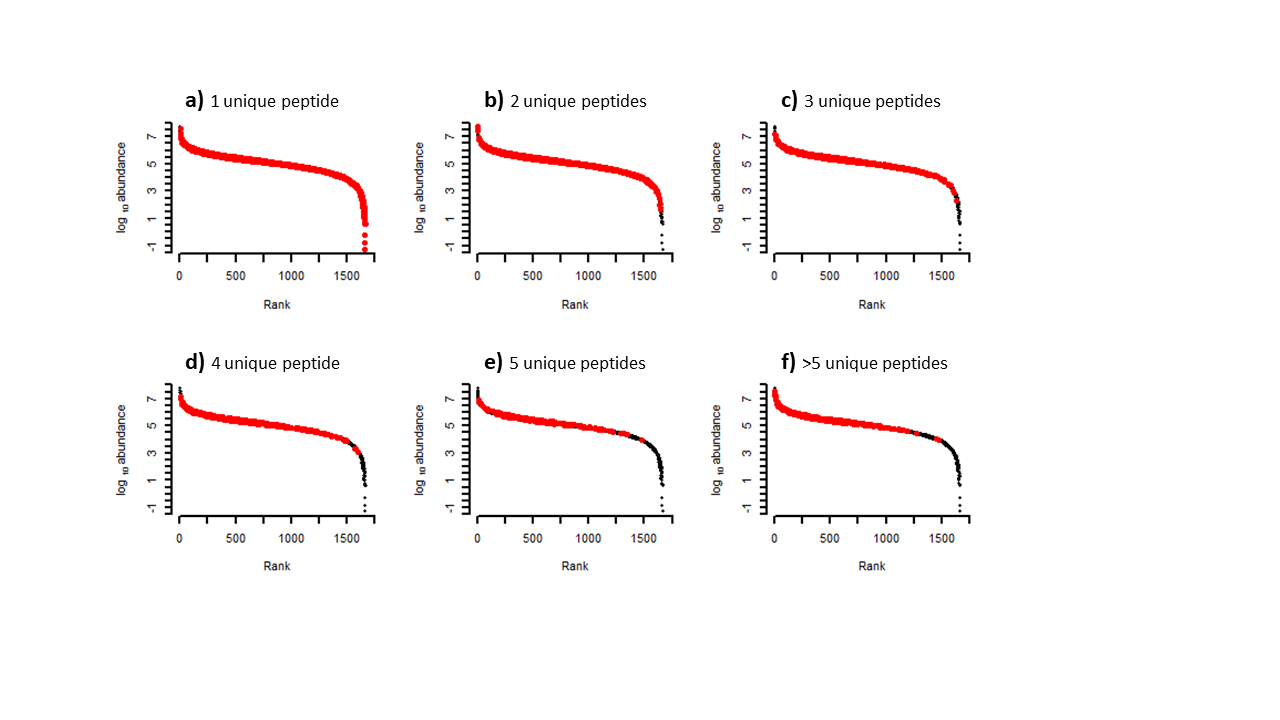

Supplement: S1 Fig — Proteins for which 1 (a), 2 (b), 3 (c), 4 (d), 5 (e), or more than 5 (f) unique peptides were detected. Proteins are arranged according to their dynamic range. Red points indicate proteins that meet the criteria of the corresponding panel. (TIF) [file pone.0227003.s001.tif]

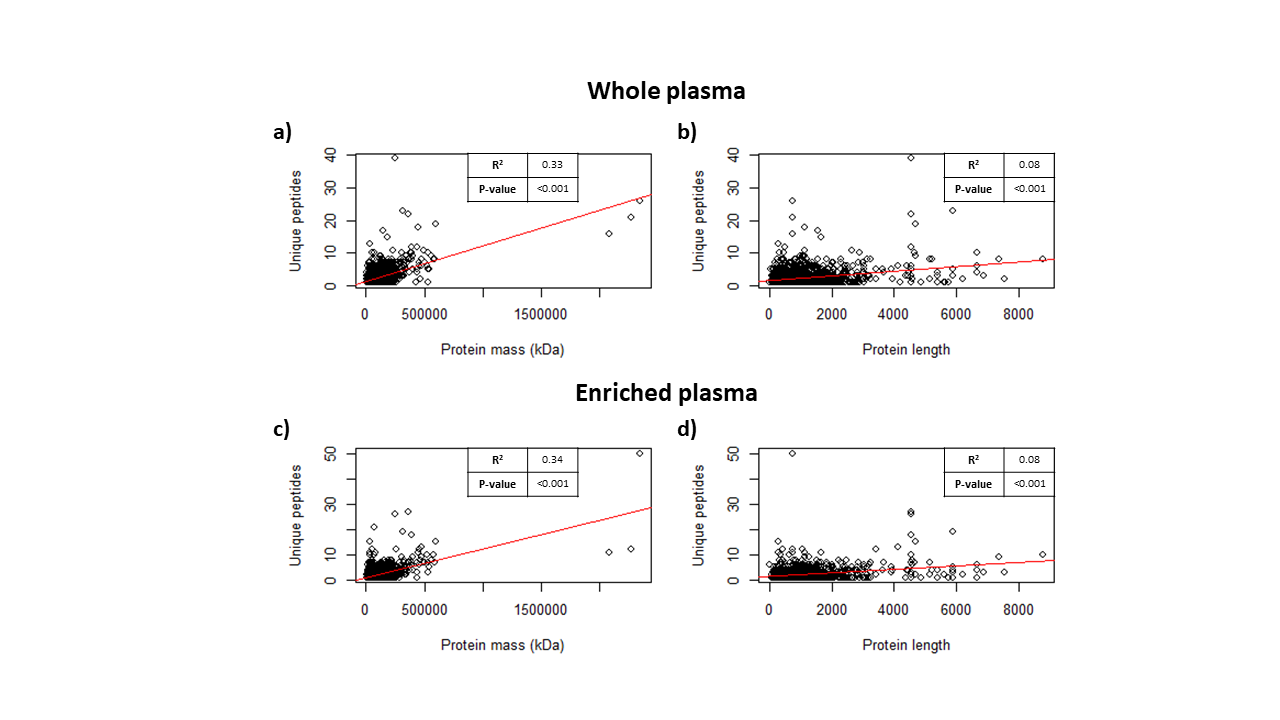

Supplement: S2 Fig — Correlation between the number of unique proteins with protein mass (a,c) and amino acid length (b,d) in whole (WP)(a,b) and in enriched plasma (EP)(c, d). (TIF) [file pone.0227003.s002.tif]

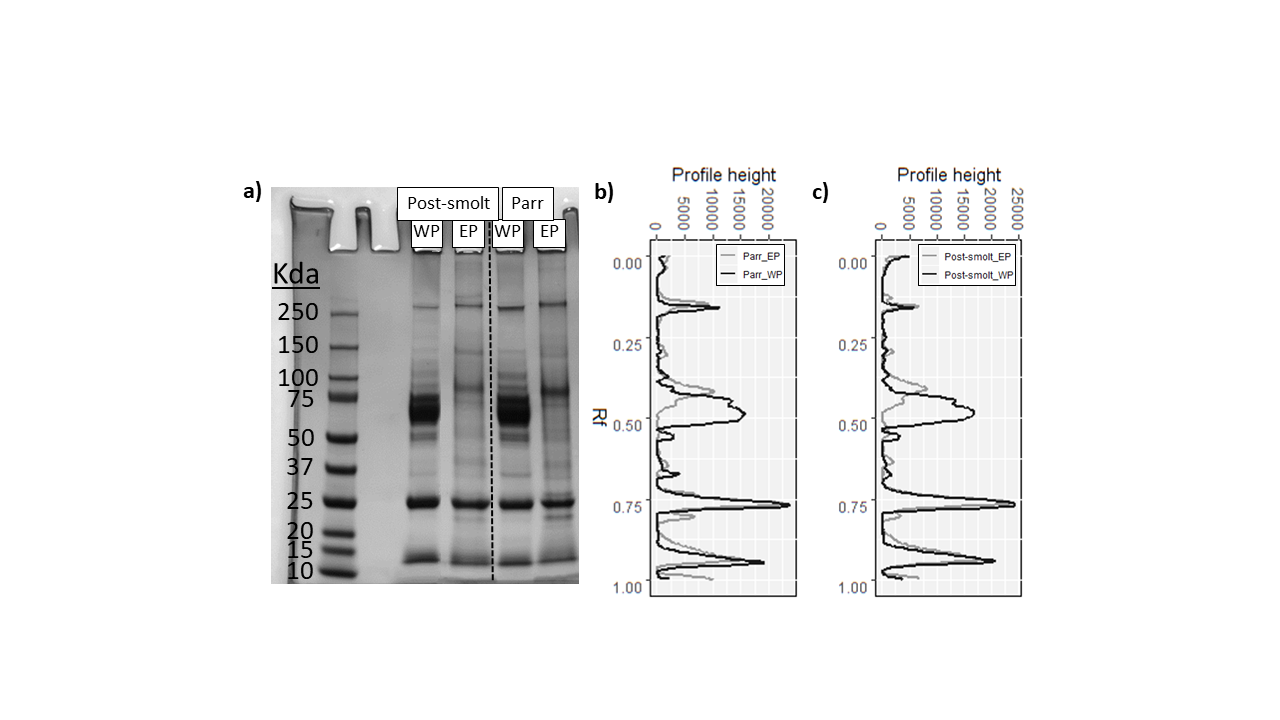

Supplement: S3 Fig — Gel image (a), densitometry of Post-smolt pool WP and EP (b) and densitometry of Parr pool WP and EP (c). Dashed line indicates where gel was edited to remove unwanted gel lanes. Original gel image is provided as S1 Raw images. (TIF) [file pone.0227003.s003.tif]

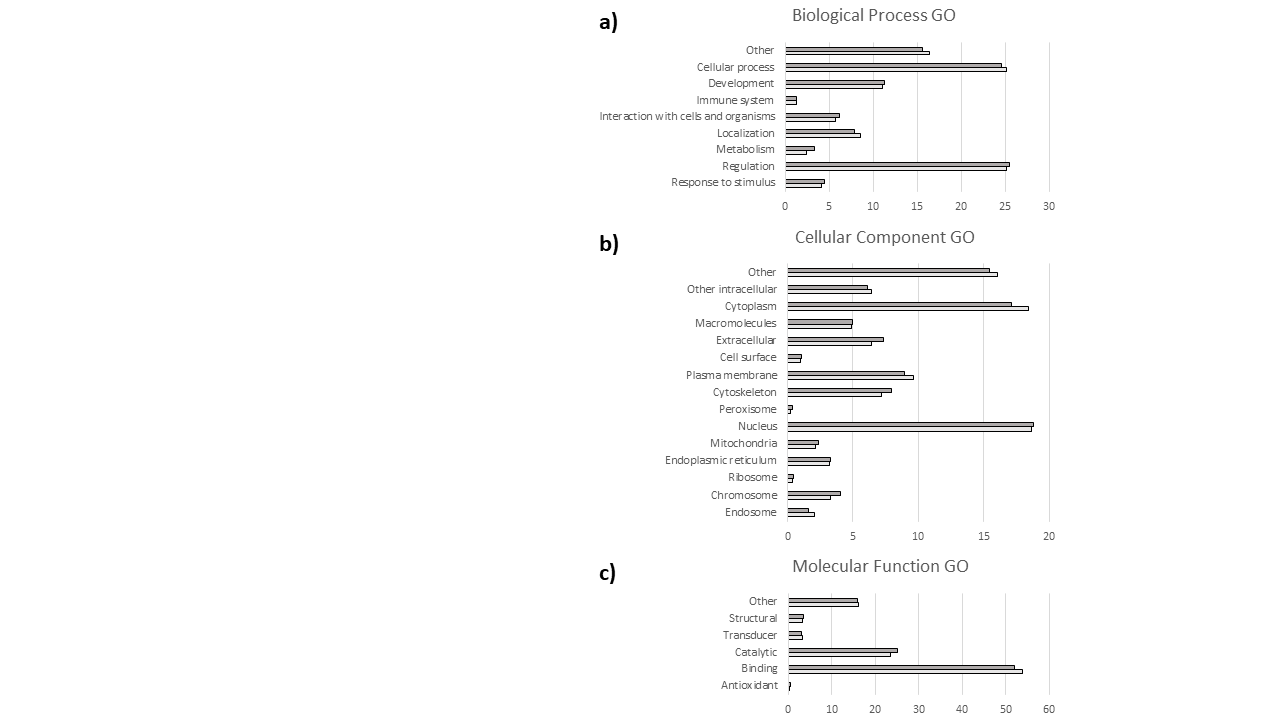

Supplement: S4 Fig — Biological GO (a), cellular component GO (b) and molecular function GO (c). (TIF) [file pone.0227003.s004.tif]

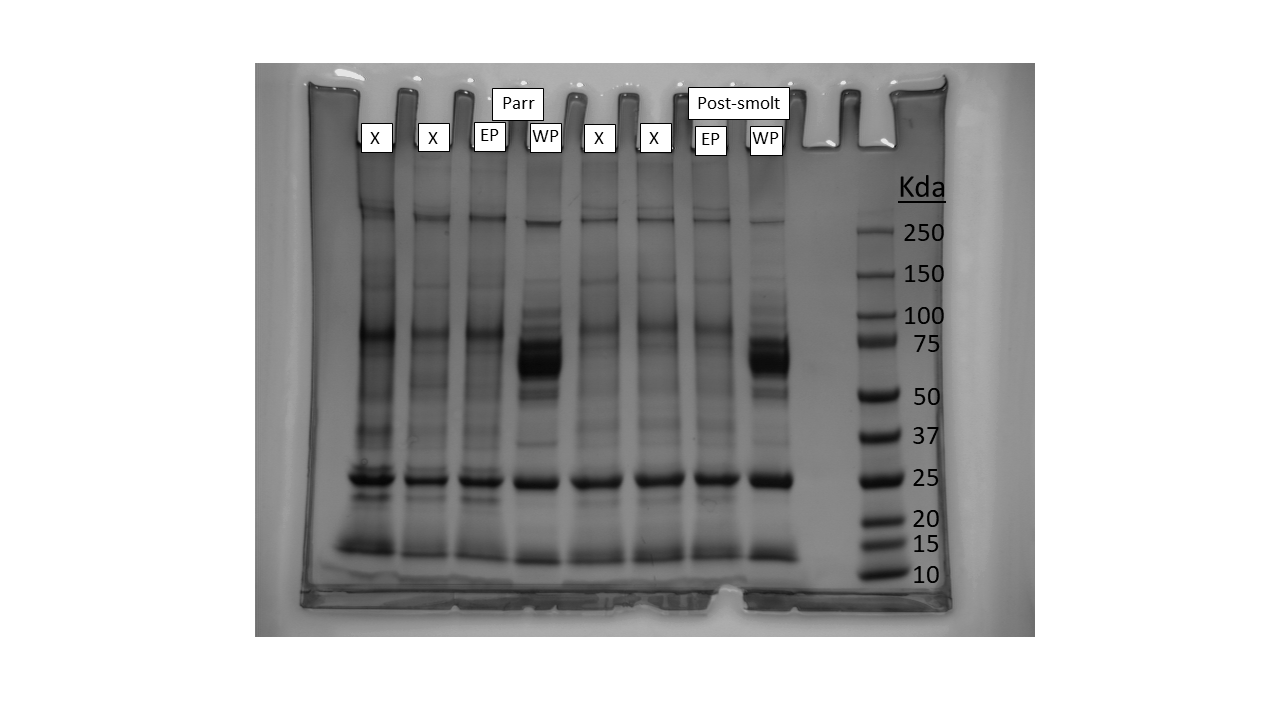

Supplement: S1 Raw images — (TIF) [file pone.0227003.s009.tif]
